# Mapping the Human Chondroitin Sulfate Glycoproteome Reveals an Unexpected Correlation Between Glycan Sulfation and Attachment Site Characteristics

**DOI:** 10.1016/j.mcpro.2023.100617

**Published:** 2023-07-14

**Authors:** Fredrik Noborn, Jonas Nilsson, Carina Sihlbom, Mahnaz Nikpour, Lena Kjellén, Göran Larson

**Affiliations:** 1 Department of Laboratory Medicine, Institute of Biomedicine, Sahlgrenska Academy, University of Gothenburg, Gothenburg, Sweden; 2 Proteomics Core Facility, Sahlgrenska Academy, University of Gothenburg, Gothenburg, Sweden; 3 Department of Medical Biochemistry and Microbiology, Uppsala University, Uppsala, Sweden; 4 Laboratory of Clinical Chemistry, Sahlgrenska University Hospital, Gothenburg, Sweden

**Keywords:** glycosaminoglycans, chondroitin sulfate, proteoglycans, glycoproteomics, glycopeptides

## Abstract

Chondroitin sulfate proteoglycans (CSPGs) control key events in human health and disease and are composed of chondroitin sulfate (CS) polysaccharide(s) attached to different core proteins. Detailed information on the biological effects of site-specific CS structures is scarce as the polysaccharides are typically released from their core proteins prior to analysis. Here we present a novel glycoproteomic approach for site-specific sequencing of CS modifications from human urine. Software-assisted and manual analysis revealed that certain core proteins carried CS with abundant sulfate modifications, while others carried CS with lower levels of sulfation. Inspection of the amino acid sequences surrounding the attachment sites indicated that the acidity of the attachment site motifs increased the levels of CS sulfation, and statistical analysis confirmed this relationship. However, not only the acidity but also the sequence and characteristics of specific amino acids in the proximity of the serine glycosylation site correlated with the degree of sulfation. These results demonstrate attachment site-specific characteristics of CS polysaccharides of CSPGs in human urine and indicate that this novel method may assist in elucidating the biosynthesis and functional roles of CSPGs in cellular physiology.

## Highlights

• Human chondroitin sulfate proteoglycans (CSPGs) vary in their levels of sulfation.
• Novel glycoproteomic analyses of CSPGs of human urine enabled site-specific sequencing of CS chains.
• The CS sulfation level correlated with the acidity of the amino acid attachment motif.
• This suggests a link between the amino acid sequence and CS biosynthesis.

## Graphical Abstract

**Figure:**
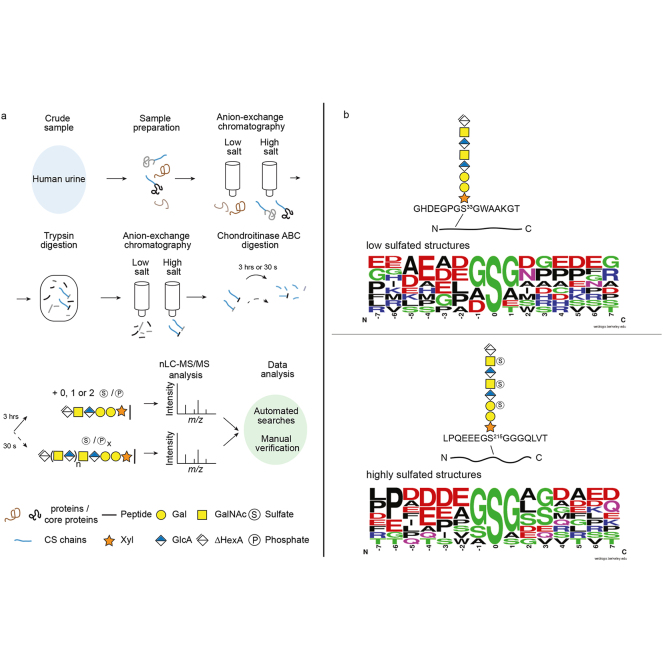

## In Brief

Using a novel glycoproteomic approach for site-specific sequencing of chondroitin sulfate (CS) proteoglycans, we found that the glycans varied considerably in their levels of sulfation. Analysis of the amino acid sequences surrounding the attachment sites indicated that the acidity of the attachment site motifs as well as the amino acid identities and positions in proximity to the serine glycosylation site correlated with the level of CS sulfation, linking the primary sequence of the core protein to glycosaminoglycan biosynthesis and modifications.

Vertebrates produce various chondroitin sulfate proteoglycans (CSPGs), ubiquitously expressed at cell surfaces and connective tissues (1). So far, more than 70 different CSPGs have been identified in humans, each distinguished by the primary amino acid sequence of the core proteins (2). The core proteins are modified with one or more CS polysaccharide chains that interact with a variety of ligands, such as growth factors, cytokines, and cell adhesion molecules (3, 4, 5). The CS–ligand interactions are dependent on structural variants of the polysaccharides, which thereby contribute to the control of key functions in development and homeostasis (6). The specificities of the interactions are greatly influenced by the levels and positions of negatively charged carboxyl and sulfate groups along the polysaccharides, thus making CS structural characterization essential for the functional understanding of CSPGs (7).

**Fig. 1:**
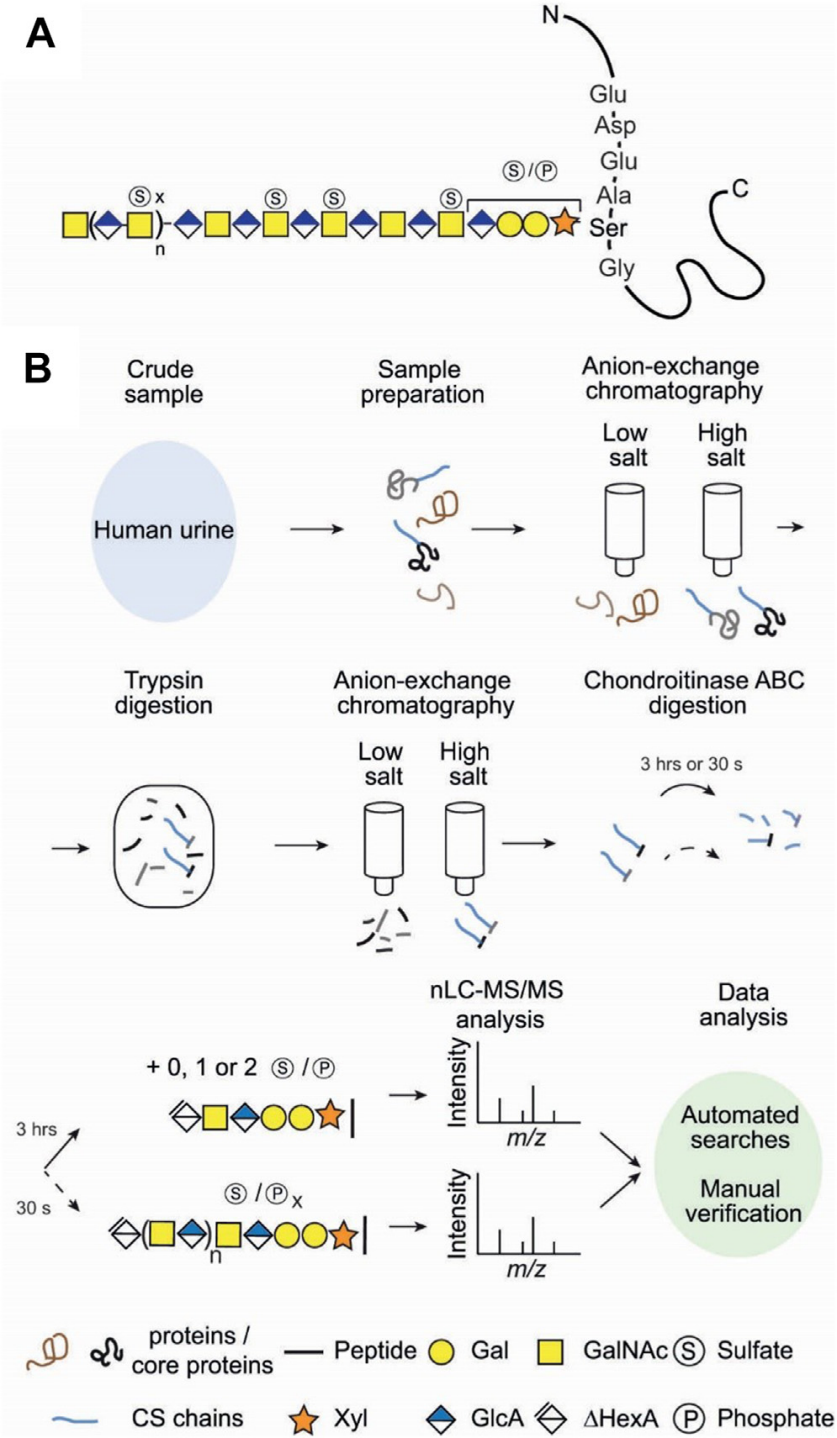
**Schematic illustration of CSPGs and the workflow used for site-specific characterization of CS glycopeptides.***A*, schematic illustration of a CS polysaccharide and an acidic consensus attachment motif. Here, the attachment motif for decorin (Glu-Asp-Glu-Ala-Ser-Gly) is shown. The number of disaccharides (n) and sulfation level (x) may vary for each CS polysaccharide and each glycopeptide of decorin. *B*, general workflow from sample preparation to nLC-MS/MS-analysis and data evaluation. After the GAG-glycopeptide enrichment steps, the preparations were depolymerized with chondroitinase ABC for either 30 s or 3 h, which generated CS structures of variable lengths. The two preparations were analyzed separately with nLC-MS/MS and the data sets obtained were evaluated with a combination of automated Mascot-searches and manual interpretations. CS structures of different lengths (n) and sulfation degree (x) were included in the searches.

The CS biosynthesis is initiated by the transfer of a xylose (Xyl)-residue to specific serine residues of the core proteins by xylosyltransferases 1 or 2 (XYLT1 and XYLT2). Owing to the specificities of the enzymes, the serine residues selected for Xyl-modification are usually flanked by a glycine (-SG-) or an alanine (-SA-) in the C-terminal direction and are sometimes also associated with acidic amino acids in close proximity (8, 9). The biosynthesis continues with the enzymatic transfer of two galactoses (Gal) and one glucuronic acid (GlcA) residue, completing the formation of the canonical tetrasaccharide linkage region (GlcA-Gal-Gal-Xyl-*O*-Ser). The linkage regions are thereafter elongated by the repeated addition of GalNAc and GlcA residues and are further modified by CS-specific sulfotransferases, generating structures with complex, yet defined, sequences. To date, six glycosyltransferases and seven sulfotransferases responsible for polymerization and sulfate modifications have been identified in humans (1, 10). A schematic illustration of a typical CS structure and a CS attachment motif is shown in Figure 1*A*.

In contrast to the synthesis of oligonucleotides and proteins, glycan biosynthesis is often regarded as a non-template-driven process (11, 12). That generally means that the glycan structures produced are determined by the temporal- and tissue-specific expressions of the biosynthetic enzymes and the availability of their substrates in a membrane-enclosed subcellular compartment (13). Based on such notion, it is assumed that CS, or other glycosaminoglycan (GAG) structures, from any given tissue or cell will be more or less identical regardless of the core proteins, or their specific serine residues, to which they are attached. However, certain reports have established that, through mechanisms that have yet to be unraveled, specific core protein determinants may indeed influence the GAG structure (14, 15, 16, 17).

To date, mass spectrometry (MS) is a well-established method for the structural characterization of the disaccharide components of CS chains. Such methods are typically based on electrospray ionization and negative ion mode analysis, thereby exploiting the acidic nature of the carboxyl- and sulfate groups of the polysaccharides (18, 19). However, CS sequencing is a challenging undertaking due to the heterogeneous population of CS chains, varying in sulfation degree, sulfation pattern, epimerization of glucuronic acid to iduronic acid, and polysaccharide chain length. Thus, specific bacterial lyases are often used in different combinations (*e.g.*, chondroitinase ABC or AC/B) to reduce the length and complexity of the CS chains, generating shorter segments such as di- and oligosaccharides that are simpler to separate and analyze (20, 21, 22). So far, sequencing of full-length CS chains has been accomplished only for two proteoglycans, both carrying a single CS chain (bikunin (11) and decorin (23)). The sequencing was achieved by separating the heterogeneous CS mixture into fractions of different chain lengths followed by negative mode MS/MS-analysis, demonstrating that full-length sequencing is indeed feasible for highly purified CSPG preparations. However, at present, there is a major technical difficulty in obtaining such structural information from core protein-specific polysaccharides from complex mixtures of CSPGs.

We have previously developed a glycoproteomic approach for the characterization of CS glycopeptides in complex tissue samples, resulting in the identification of novel core proteins in human and animal models (2, 4, 24, 25, 26). The identification is achieved by trypsin digestion of CSPG-containing samples, followed by CS glycopeptide enrichment using strong-anion-exchange (SAX) chromatography. The enriched glycopeptides are then incubated with chondroitinase ABC to depolymerize the CS polysaccharides, generating hexasaccharide (6-mer) residual structures including the linkage regions still attached to the peptides. The preparations are analyzed by nano-scale reversed-phase liquid chromatography tandem mass spectrometry (nLC-MS/MS) in positive mode, and the data are evaluated through proteomic database searches with adjustments in the settings to allow for glycopeptide identification.

Here, we further develop this concept to achieve attachment site-specific sequencing of longer residual CS structures. CS glycopeptides were enriched from human urine and digested at different times with chondroitinase ABC, yielding peptide-attached CS structures of 6 to 18 monosaccharides in length. Structural analysis typically revealed core protein-specific CS structures, where some core proteins carried CS with little or no sulfation, whereas others contained CS with abundant sulfation toward the reducing end of the chains. Inspection of the amino acid sequence for each core protein indicated that the level of sulfation correlated with the acidity of the attachment motifs, an observation that was subsequently confirmed by statistical analysis. This result would offer an explanation to earlier findings of the apparent influence of the core protein on CS structure and provides a theoretical framework for investigating novel regulatory aspects of CS biosynthesis. Furthermore, this method may also assist in delineating structural-functional relationships of CSPG-dependent biological functions and in biomedical research.

## Experimental Procedures

### Preparation of CS-Glycopeptides

Human urine samples were prepared in line with the previously described protocol, with some modifications (24). The use of de-identified human samples for method development is in agreement with Swedish law and was formally permitted by the head of the Laboratory of Clinical Chemistry, Sahlgrenska University Hospital. The research involving human participants was conducted in compliance with the Helsinki Declaration.

Briefly, 25 ml of morning urine was centrifuged (2000*g* for 5 min) to remove cell debris and was thereafter diluted in 250 ml coupling buffer (50 mM NaAc, 200 mM NaCl, pH 4.0). Twenty-five ml of diluted sample was applied onto a SAX-chromatography column (Vivapure, Q Maxi H; bead volume 2700 μl), and the column was spun at 2000*g* for 1 min. The procedure was repeated until all sample volume had been applied onto the column. The column was thereafter washed with 15 ml 50 mM Tris-HCl, 200 mM NaCl, and pH 8.0 to remove loosely bound material. The proteoglycans were eluted with 5 ml of 2 M NaCl, pH 8.0, and the column was spun for 4 min at 4000*g*. The collected fraction was desalted with a PD10 column, lyophilized, and then subjected to an in-solution trypsin digestion protocol, as previously described (24). The digested sample was diluted in 25 ml coupling buffer and applied to a second SAX-chromatography column to enrich for GAG-substituted peptides, according to the above-described procedure. The eluted fraction was desalted and then treated with 10 mU of chondroitinase ABC (Sigma-Aldrich) in 50 μl digestion buffer (55 mM NH_4_OAc, pH 8.0) where half of the material was treated for 30 s and the other half for 3 h, respectively.

In another experiment, full-length bikunin CS-glycopeptides were enriched from human urine. As bikunin is highly abundant in human urine, a single SAX step is sufficient to enrich for bikunin glycopeptides. 10 milliliter of the same pool of human urine was digested with trypsin using an in-solution protocol and the sample was thereafter enriched for CS glycopeptides with SAX-chromatography (Vivapure, Q-mini H), as described previously (24). The bound material was eluted with a single fraction of high salt concentration (1.6 M NaCl), which was desalted and then subjected to HS depolymerization. The sample was incubated with a mix of 5 mU heparinase II (no EC number) and 5 mU heparinase III (EC 4.2.2.8) (both from *Pedobacter heparinus* overexpressed in *Escherichia coli*; kind gift from Prof. Jian Liu, University of North Carolina) in HS digestion buffer (50 mM NH_4_OAc, 4 mM CaCl_2_, pH 7.3).

### LC-MS/MS Analysis in Positive Mode

The samples were analyzed on an Orbitrap Fusion mass spectrometer coupled to an Easy-nLC 1200 liquid chromatography system (both Thermo Fisher Scientific). Glycopeptides (3 μl injection volume) were trapped on an Acclaim PepMap 100 C18 trap column (100 μm × 2 cm, particle size 5 μm, Thermo Fischer Scientific) and separated on an analytical column (75 μm × 30 cm) packed in-house with Reprosil-Pur C18 material (particle size 3 μm, Dr Maisch). The gradient was run at 300 nl/min; 7 to 35% B-solvent over 45 min, 35% to 100% B over 5 min, with a final hold at 100% B for 10 min; solvent A was 0.2% formic acid (FA) in water, solvent B was 80% acetonitrile, 0.2% FA. The analysis of full-length CS glycopeptides was run with a similar gradient as above with some minor changes: 10% to 40% B-solvent over 60 min, 40% to 100% over 5 min with a final hold at 100% B for 10 min.

Nanospray Flex ion source was operated in positive ionization mode at 1.8 kV. MS scans were performed at 120,000 resolution (at *m/z* 200), with an Automatic Gain Control (AGC)-target value of 1E6, maximum injection time of 50 ms, and scan range of *m/z* 600 to 2000. The most abundant precursor ions with charges from 2+ to 7+ were selected for fragmentation with a maximum cycle time of 3 s and dynamic exclusion with a duration of 10 s. Three separate higher-energy collision-induced dissociation (HCD) MS/MS spectra were recorded for each precursor: scan 1 at the HCD collision energy of 30% with the scan range from *m/z* 100 to 2000, scan 2 at the HCD energy of 40% with the scan range from *m/z* 300 to 2000, and scan 3 at the HCD energy of 40% with the scan range from *m/z* 100 to 2000. All MS/MS scans were acquired with the precursor isolation window of 5.0, resolution 30,000 (at *m/z* 200), AGC target of 1e5, and maximum injection time of 118 ms. The analysis of full-length CS glycopeptides was run with similar settings as above, with some minor modifications: maximum injection time 100 ms, MS/MS scans were acquired with the precursor isolation window of 2.5, resolution 30,000 (at *m/z* 200), first *m/z* 100, AGC target of 5e4 and maximum injection time of 75 ms. MS/MS spectra were recorded for each precursor with the collision energy at 20%, 30%, and 35%.

### Data Analysis

The files were analyzed for CS glycopeptides using both manual interpretation and automated Mascot database searches. The Xcalibur software (Thermo Fisher Scientific) was used for manual interpretation. Database searches were performed against *Homo sapiens* in the UniProtKB/Swiss-Prot database (20,431 sequences, 06/05/2019) using Mascot Distiller (version 2.6.1.0, Matrix Science) and an in-house Mascot server (version 2.5.1). The Mascot searches employed the criteria *Trypsin* as enzyme specificity (cleavage after Lys and Arg), allowing for up to two missed cleavages. Subsequent searches were performed, allowing for a non-trypsin cleavage end/start residue (*Semitrypsin*). The peptide tolerance was 5 ppm and the fragment tolerance 20 ppm. Initially, searches were made for CS 6-mer structures at serine residues, with varying degrees of modifications, including the 6-mer structure [HexUA(-H_2_O)-HexNAc-HexUA-Hex-Hex-Xyl-O-] with 0 (C_37_H_55_NO_30_, 993.2809 u), 1 (C_37_H_55_NO_33_S, 1073.2377 u), or 2 (C_37_H_55_NO_36_S_2_, 1153.1945 u) sulfate groups attached. For each of the modification entries, the same mass was also added as a required neutral loss with *Searching* specified. Additional searches were made for extended structures, including octa-, deca-, and dodecasaccharides, with varying degrees of sulfation, varying from unmodified to abundantly sulfated structures (maximum n + 2 sulfate groups, where n is the number of GalNAc residues in the glycan). The modifications were constructed by adding additional disaccharide unit(s) [HexUA-HexNAc (379.1115 u)] and sulfate group(s) [SO_3_H (79.9568 u)] to the initial 6-mer structure. The Mascot-generated hits were manually evaluated to identify potential false-positive hits according to previously described criteria (27). Briefly, the hits were manually verified to ensure the presence of HexNAc-oxonium ions, *m*/*z* 362.11 [ΔHexA–H_2_O+HexNAc]^+^ and *m*/*z* 214.09 [HexNAc+CO–H_2_O]^+^, which are diagnostic for the linkage region 6-mer structures. The hits were manually verified to also display stepwise glycosidic fragmentation of the linkage region and/or the peak(s) corresponding to the de-glycosylated peptide ion. Moreover, the hits were verified to contain at least three b- and y-ions to ensure that the peptide sequences encompassed the correct start and end residues. The Mascot-generated hits that did not conform to all the above-stated criteria were discarded as false-positive hits. Furthermore, in the verification of glycopeptide identities, MS1 spectra were also investigated to ensure the absence of any significant unrelated precursor ions within the isolation window.

### Experimental Design and Statistical Rational

In this study, we used two different urine samples collected from two healthy control individuals. The first urine sample was enriched for GAG-glycopeptides and the preparation was divided into two parts and was either depolymerized with chondroitinase ABC for 3 h to generate residual 6-mer structures or 30 s to generate longer residual structures of varying lengths. The results of the 3 hrs-experiment showed very good overlap with previous experiments (24), thus providing a biological and methodological reference control. The identified CS-proteoglycans in the 30 s-experiment also showed good overlap with the 3-hrs-experiment (although the total number of identified CS-proteoglycans was fewer, which is expected as the longer chains give weaker signals). A second urine sample was also enriched for GAG-glycopeptides but was not treated with chondroitinase ABC to enable the identification of intact CS glycopeptides. The preparation was instead treated with heparinase to degrade any co-enriched HS-glycopeptides, which may otherwise complicate the analysis. Statistical analysis of the correlation between the number of sulfates, and the acidity of the attachment motifs was performed using Graphpad Prism (version 8.0). The theoretical isoelectric point (pI) was calculated for the attachment motif (−5 to +5 amino acid residues from the attachment site) of the identified core proteins using ProtParam, ExPASy (28). For sequences containing more than one serine residue, which implies some ambiguity of the exact attachment site, the most probable serine residue was assigned based on the general notion of GAG attachment motifs (9, 29). In cases with several serine residues, the following rule was applied: GSG > SG > GS/SA/AS > other variants. The glycopeptide hits of 6-mer and 8-mer structures were sorted into three different groups depending on their number of sulfate modifications. The number of glycopeptide hits was based on spectral counting of annotated MS/MS spectra from the Mascot-assisted analysis and constituted a total of 255 spectra for the 6-mer glycopeptides, and 51 spectra for the 8-mer glycopeptides, respectively. For the 6-mer glycopeptides, the hits were grouped into 0, 1, and 2 sulfate modifications, respectively. For the 8-mer glycopeptides, the hits were grouped into 0 to 1, 2, and 3 to 4 sulfate modifications, respectively. One-way ANOVA tests were used for statistical analysis. Statistical analyses of aligned CS attachment sites in humans were performed using Weblogo (30).

## Results

### Enrichment and nLC-MS/MS-Analysis of CS-Glycopeptides

Human urine was chosen as a sample matrix as it is biomedically relevant and contains several different CSPGs and thus is a suitable system for attachment site-specific CS structural analysis (24). A two-step SAX enrichment procedure was developed to isolate CS glycopeptides in larger quantities from human urine. In the first step, the proteoglycans were enriched by an anion-exchange column to remove contaminating proteins. The enriched proteoglycans were then digested with trypsin and passed over a second anion-exchange column to enrich for anionic GAG glycopeptides and to remove non-modified peptides. The enriched GAG glycopeptides were then incubated with chondroitinase ABC for 3 h, followed by nLC-MS/MS analysis. The 3 h chondroitinase incubation results in exhaustive degradation of the CS chains, generating residual 6-mer structures still attached to the peptides, composed of the linkage region and a terminal unsaturated ΔHexA-GalNAc disaccharide. In agreement with our previous reports using this protocol for the characterization of CSPGs (24, 25) (Fig. 1*B*), incubation with chondroitinase ABC for 3 h enabled the identification of multiple CS glycopeptides carrying residual 6-mer structures. Proteomic database searches were designed to include variable modifications of the residual 6-mer structure (993.2808 u) for [HexA(-H_2_O)GalNAcGlcAGalGalXyl-*O*-] with 0, 1, or 2 sulfates or phosphates attached, and all hits were manually validated and interpreted with regard to peptide sequence, glycan structure, and precursor mass. In total, the glycoproteomic analysis identified 21 previously established CSPGs as well as five novel CSPGs (2, 31), including membrane-associated progesterone receptor component 2 (O15173), transmembrane protein 119 (Q4V9L6), low-affinity immunoglobulin epsilon Fc receptor (P06734), polypeptide N-acetylgalactosaminyltransferase 2 (Q10471) and matrix extracellular phosphoglycoprotein (Q9NQ76) (supplemental Fig. S1, *A*–*E*). As expected, all the MS/MS spectra of the identified CS glycopeptides displayed the prominent diagnostic oxonium ion at *m/z* 362.1, which corresponds to the terminal unsaturated disaccharide [HexA(-H_2_O)GalNAc+H] ^+^ as well as unique peptide fragments for each amino acid sequence.

### Site-Specific Analysis of Residual CS Structures of 8, 10, and 12 Monosaccharides in Length

**Fig. 2:**
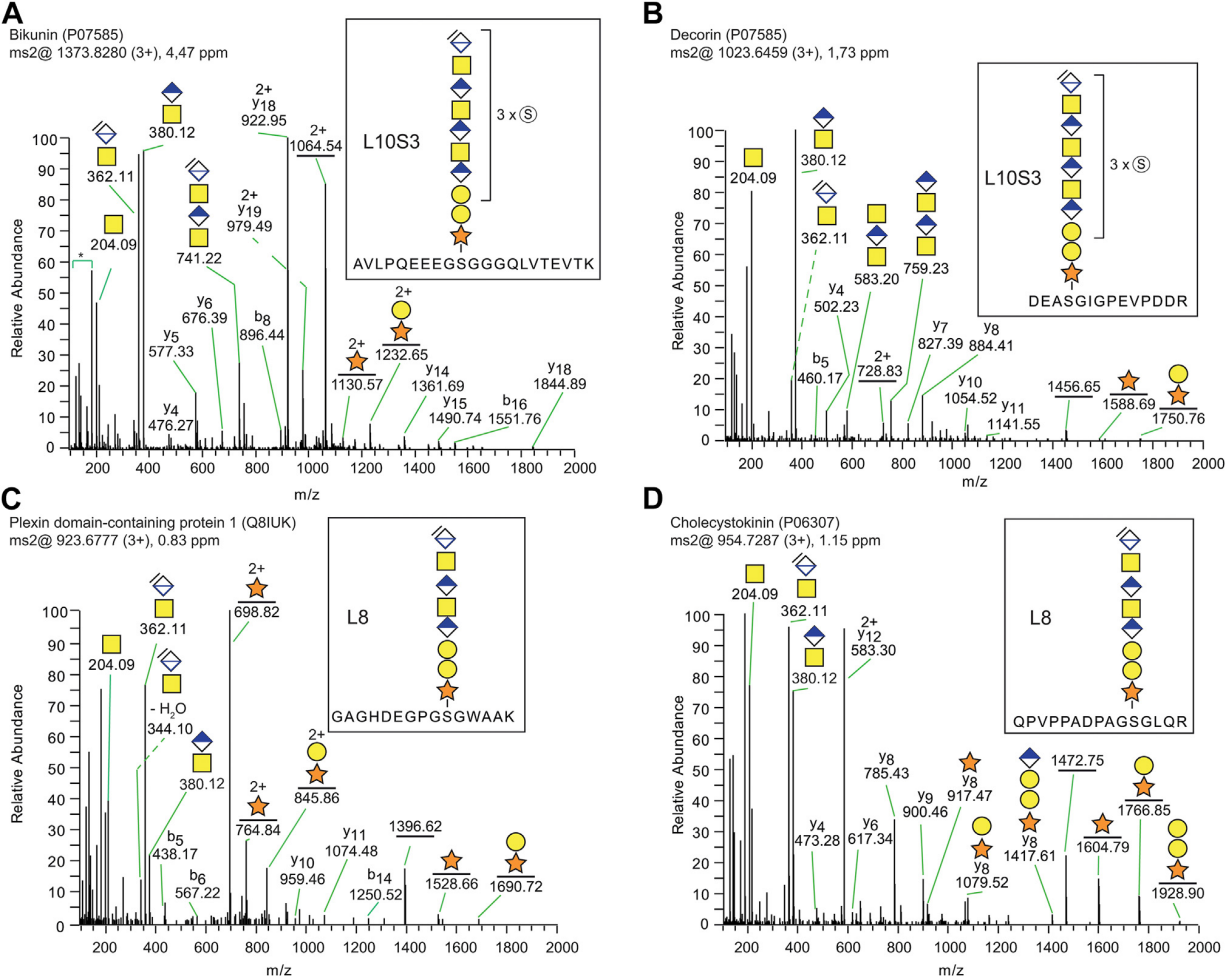
**Site-specific analysis of residual decasaccharide (10-mer) and octasaccharide (8-mer) CS structures attached to human urinary core proteins.***A*–*D*, MS/MS fragment mass spectra from four glycopeptides of different core proteins with CS structures varying in their levels of sulfation. *A*, mMass spectra of bikunin (P02760) glycopeptide (*m/z* 1373.8280; 3+) and (*B*) decorin (P07585) glycopeptide (*m/z* 1023.6459; 3+) both show 10-mer residual structures with three sulfate modifications. In contrast, mass spectra of (*C*) plexin domain-containing protein-1 (Q8IUK5) glycopeptide (*m/z* 923.6777; 3+) and (*D*) cholecystokinin (P06307) glycopeptide (*m/z* 954.7287; 3+) show 8-mer residual structures without any sulfate modifications. The analysis also enabled the identification of several GalNAc-derived oxonium ions in the low mass range in all of these spectra although marked here only with an “∗” in panel 2a, (see also supplemental Fig. S2).

We then tested if we could also generate longer residual structures (*i.e.*, 8-, 10-, 12-monosaccharide residues) by incubating enriched GAG glycopeptides with chondroitinase ABC for a shorter period of time (30 s), to only partially depolymerize the CS chains. The general workflow for GAG glycopeptide generation and enrichment, the different chondroitinase depolymerization procedures (3 h and 30 s), and the subsequent MS/MS-analyses are shown in Figure 1*B*. To investigate whether proteomic database searches could identify also extended residual structures, the searches were allowed to include variable modifications of 8-, 10-, and 12-mer oligosaccharides, all with varying levels of sulfation. Interestingly, the automatic searches identified several CS glycopeptides with longer residual structures: eight different core proteins were found with residual CS structures of eight or more monosaccharide residues in length (supplemental Table S1a). In Figure 2, examples of MS/MS spectra from four different core proteins (bikunin, decorin, plexin domain-containing protein 1 and cholecystokinin) carrying residual 8- and 10-mer structures are shown. Of note, the spectra contained several diagnostic glycan fragments which are derived from different parts of the glycan chain. In addition to the prominent ion at *m/z* 362.1, the glycopeptide spectra of 8-mer or longer CS structures also contained the fragment ion at *m/z* 380.1, that is, [HexAGalNAc+H]^+^ ion, which when appearing together with the *m/z* 362.1 ion is diagnostic for internal non-dehydrated disaccharides and would be expected from 8-mer and longer CS structures. Furthermore, the analysis also enabled the identification of several GalNAc-derived oxonium ions in the low mass range (supplemental Fig. S2) (24). In keeping with earlier structural studies (11, 23), the bikunin- and decorin-associated CS structures were consistently found with two or more sulfates, demonstrating that these core proteins carry CS structures with a relatively high level of sulfation (supplemental Table S1, a and b). Figure 2, *A* and *B* shows MS/MS spectra of CS glycopeptides of bikunin and decorin with residual 10-mer structures, where each precursor ion was modified with three sulfate groups. Since assigning the exact monosaccharide positions was difficult due to the low intensities of sulfate-related fragment ions in MS/MS, the determination of sulfate modification was based on the equating mass of the precursor ion with the theoretical structure (<10 ppm). Of note, in contrast to the abundant sulfate modifications of bikunin and decorin, other core proteins carried structures with no or only a few sulfate groups. For instance, plexin domain-containing protein 1 carried mainly 10-mer structures with a lower level of sulfation (0 or 1 sulfate group), while cholecystokinin was exclusively found without any sulfate modifications at all (Fig. 2, *C* and *D*) (supplemental Fig. S3) (supplemental Table S1a), demonstrating that the level of sulfation may vary depending on the core protein in human urine.

Interestingly, the detailed evaluation revealed that some neuropeptide W- and bikunin-linked oligosaccharides (8-mer and 10-mer structures) carried a phosphate modification on their Xyl residues (supplemental Fig. S4, *A*–*D*). These findings of longer CS structures still carrying Xyl-phosphate modifications were surprising, as Xyl dephosphorylation is considered to occur immediately after the completion of the linkage region and just before the initiation of GAG polymerization (32). The distinction between phosphates and sulfates was feasible by examining the MS/MS spectra in a narrower mass range. For example, a mass shift of 212.0064 u was observed between the neuropeptide W-derived non-modified peptide (*m/z* 985.9775; 2+) and the glycopeptide (*m/z* = 1091.9807; 2), which corresponds to the combined mass loss of a Xyl and a phosphate group with high accuracy (−10.4 ppm) [Xyl (132.0423 u) + phosphate group (79.9663 u) = 212.0086 u] (supplemental Fig. S4*C*). This accuracy is in much better agreement than a supposed sulfate modification on the Xyl residue (−44.8 ppm) [Xyl (132.0423 u) + sulfate group (79.9663 u) = 211.9991 u]. Also, the bikunin-linked oligosaccharides with one sulfate and one phosphate modification in the linkage region separated chromatographically from that with two sulfate modifications (supplemental Fig. S5, *A*–*C*).

### Site-Specific Analysis of Glycopeptides With Extended Depolymerized and Full-Length CS Polysaccharides

**Fig. 3:**
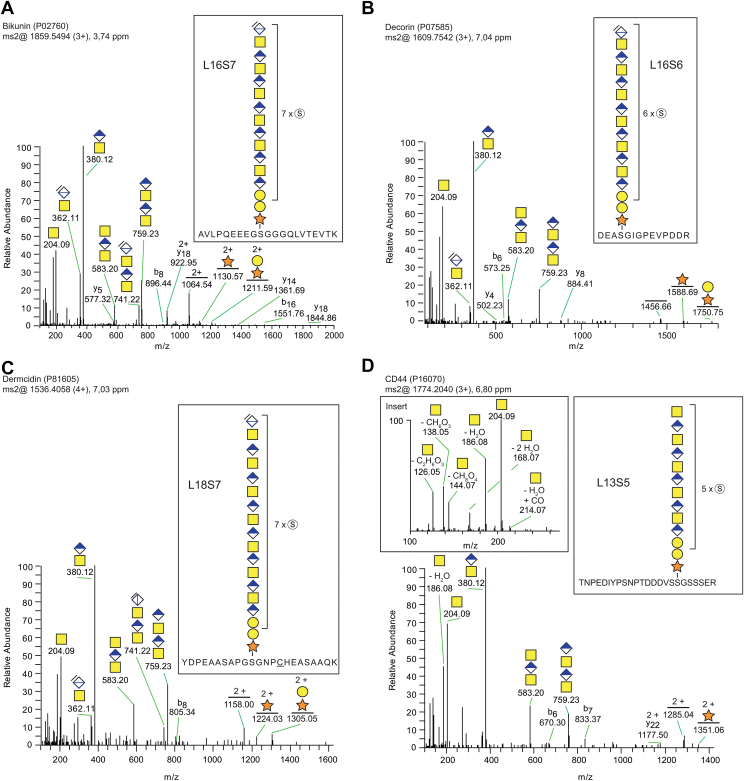
**Site-specific analysis of residual CS structures attached to human urinary core proteins.***A*–*D*, short chondroitinase ABC incubation (30 s) generated longer residual CS structures. *A*, MS/MS spectra of bikunin (P02760) glycopeptide (*m/z* 1859.5494; 3+) and *B*, decorin (P07585) glycopeptide (*m/z* 1609.7542; 3+) showing 16-mer residual structures with seven and six sulfate modifications, respectively. *C*, MS/MS spectrum of dermcidin (P81605) glycopeptide (*m/z* 1536.4058; 4+), showing an 18-mer residual structure with seven sulfate modifications. This glycopeptide was also modified with one carbamidomethyl modification. *D*, MS/MS spectrum of CD44 (P16070) glycopeptide (*m/z* 1774.2040; 3+), showing a thirteen monosaccharide long structure with five sulfate modifications. Notably, this structure terminates with a sulfated non-dehydrated GalNAc-residue, indicating that the oligosaccharide represents an intact, full-length structure. *D, insert*, examination of the fragment ions in the low mass range showed high intensities of the GalNAc-derived oxonium ions *m/z* 126.06 and *m/z* 144.07, providing additional support of the presence of a CS-glycopeptide (as opposed to an HS-glycopeptide).

In order to also investigate the sulfate distribution on longer chains, the data were further manually examined to identify the maximal lengths observed of the glycan chains. Examination of MS1-data revealed CS chains of glycopeptides from different core proteins, which varied both in length and level of sulfation. The longest CS chains of bikunin and decorin were 16-mers, with seven and six sulfate modifications, respectively (Fig. 3, *A* and *B*). Further analysis of bikunin- and decorin-linked CS chains in the range from 10- to 16-mers showed a stepwise increase in sulfation with one additional sulfate group per GalNAc-residue (supplemental Fig. S6, *A*–*F*), suggesting a uniform sulfate distribution along the polysaccharide. Such stepwise increase in sulfation per GalNAc-residue was also seen for dermcidin-linked CS chains, which were found in the range of 8- to 18-mers (Fig. 3*C* and supplemental Fig. S7, *A*–*C*). An 18-mer with seven sulfates was observed, which gives an average of one sulfate group per GalNAc-residue (Fig. 3*C*). Collectively, these data suggest that dermcidin has a similar sulfation pattern as that of bikunin and decorin. supplemental Table S1b shows all unique CS-glycopeptide hits identified from all proteoglycans in the MS data, with either Mascot-assisted analysis or manual interpretation.

Interestingly, a CD44-derived glycopeptide modified with a CS chain terminated with an intact GalNAc residue was observed (Fig. 3*D*). This GalNAc residue at the non-reducing end precludes that any chondroitinase ABC has acted upon on the structure, implicating that this represents an intact, full-length, CS polysaccharide. Furthermore, the spectrum also lacked the diagnostic chondroitinase-related oxonium ion at *m/z* 362.1 ion (corresponding to the terminal dehydrated disaccharide [HexA(-H_2_O)GalNAc+H]^+^), corroborating the notion that this is indeed an intact CS chain (Fig. 3*D*). In addition, a detailed examination of the ion profile in the low mass range (*m/z* 100–250) revealed several HexNAc-derived oxonium ions. Such ion profiles can indeed be used for saccharide identification and to differentiate between GalNAc- and GlcNAc-residues (24). The spectra showed relatively high intensities for the GalNAc-derived ions of *m/z* 126.06 and *m/z* 144.07, thus further demonstrating the presence of a CS chain (and not an HS chain). Of note, the structure was composed of a 13-mer with five sulfate groups, suggesting that the full-length CS chains in some cases are relatively short. Moreover, the chain displayed an average of one sulfate per GalNAc residue, which is similar to that seen for bikunin, decorin, and dermcidin.

We also wanted to investigate if even longer intact CS glycopeptides could be identified. GAG glycopeptides were enriched from human urine by an anion-exchange chromatography step similar to what was described earlier, but the enriched fractions were not treated at all with chondroitinase ABC. Instead, heparinase was used to degrade any co-enriched HS-glycopeptides, which may otherwise complicate the analysis. Manual interpretation enabled the identification of a bikunin-linked CS chain (*m/z* 1385.7734; 6+) representing a 31-mer structure with five sulfate modifications (L31S5) and one water adduct (L31S5 + H_2_O). Several additional variants were observed but their exact identity could not be determined due to the considerable structural heterogeneity of the full-length structures (supplemental Fig. S8, *A*–*D*). However, the results indicate that a brief or excluded chondroitinase ABC incubation, complemented with heparinase depolymerization as used here, significantly reduces the complexity of the mass-spectrometric data while still providing enough information to enable site-specific analysis of extended CS polysaccharides.

### Correlation Between the Acidity of the Attachment Motif and the Degree of Sulfation

**Fig. 4:**
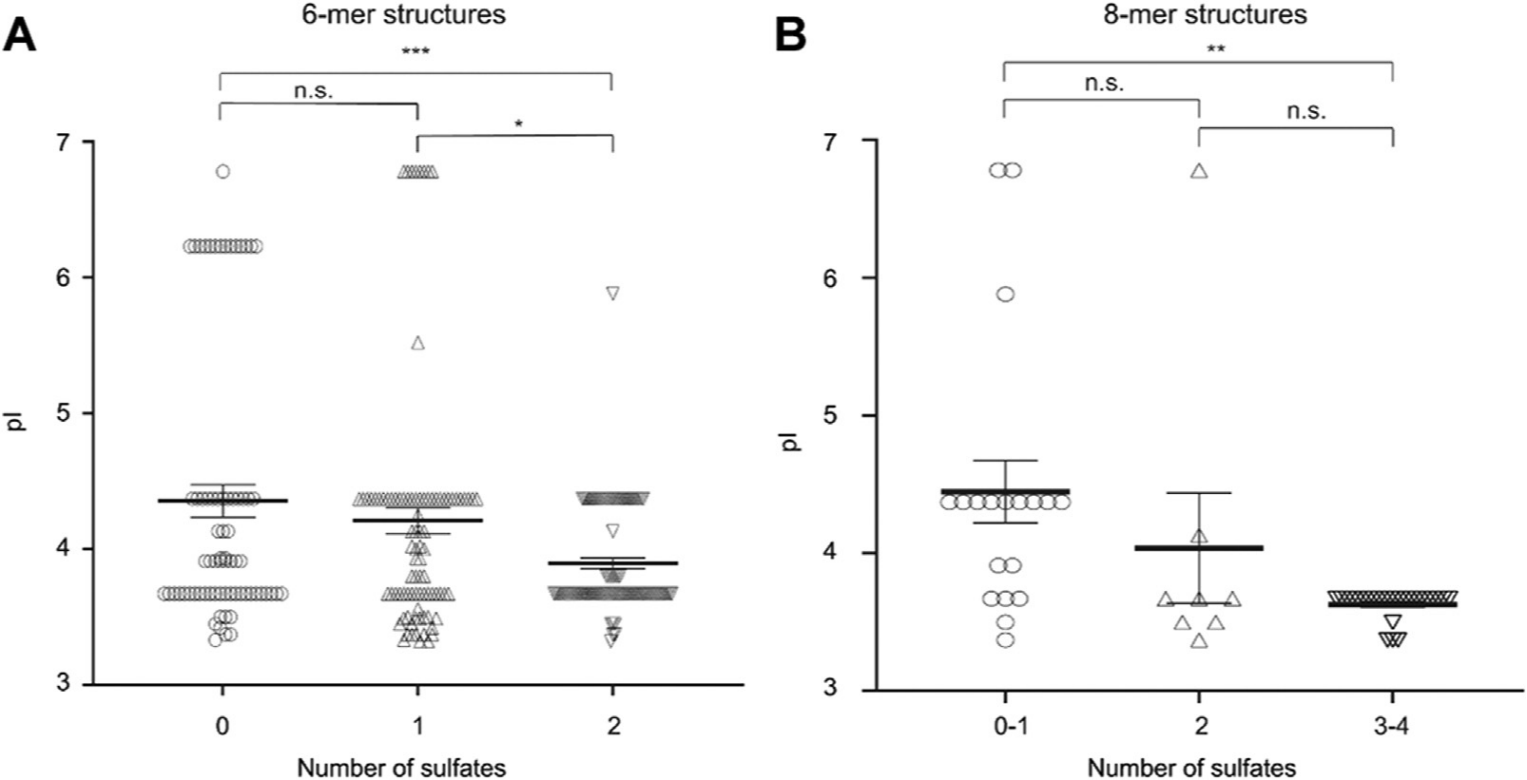
**Significant correlations between the acidities of the attachment site motifs of urinary core proteins and the levels of sulfation or phosphorylation in their CS chains.** Scatter plots illustrate the levels of sulfation/phosphorylation *versus* the isoelectric points (pI) of the attachment site motifs (−5 to +5 residues from the modified Ser) for (*A*) hexasaccharide (6-mer) and (*B*) octasaccharide (8-mer) CS structures, respectively. *Black lines* indicate mean values and error bars ± SEM. Ordinary one-way ANOVA analyses showed significant differences in pI values for both 6-mers (*A*) *p* = 0.0007 and 8-mers: (*B*) *p* = 0.0023. Tukey’s multiple comparisons test for determining statistical differences between groups gave for 6-mers (*A*) 0 *versus* 2 sulfate groups *p* < 0.001 (∗∗∗), 1 *versus* 2 sulfate groups *p* < 0.05 (∗), 0 *versus* 1 sulfate group not significant differences (n.s.). and for 8-mers (*B*) 0 to 1 *versus* 3 to 4 sulfate groups *p* < 0.01 (∗∗), 0 to 1 *versus* 2 and 2 *versus* 3 to 4 sulfate groups not significant differences in pI values. The total number of observations were 258 in (*A*) and 58 in (*B*).

Interestingly, an inspection of the glycopeptide amino acid sequences indicated that core proteins with several acidic residues around the CS glycosylation site carried more sulfated structures in their linkage regions and their immediate extensions (*e.g.*, for bikunin and decorin). In contrast, core proteins with fewer acidic residues around their attachment sites appeared to carry less sulfated structures, suggesting that the acidity of the attachment motif influences the level of sulfation. This observation is also in line with the previous notion that the initiation of GAG assembly is influenced by acidic residues in “close proximity” to the glycosylation site (8). To test the hypothesis of whether acidity influences the degree of sulfation, the isoelectric point (pI) was calculated for the attachment motif (−5 to +5 residues from the attachment site) of each core protein (supplemental Table S2) and plotted against the sulfation degree of its corresponding glycan structures. Only CS-glycopeptides with 6 and 8 sugar residues were used for the analysis in order to get comparable data from as many CSPGs as possible within each group. To assess the abundance of each glycoform, spectral counts of annotated MS/MS scans from the proteomic database searches were used and indicated that certain core proteins (*e.g.*, cholecystokinin and secretogranin-1) were always found without sulfates while others consistently had varying levels of sulfation (*e.g.*, bikunin and decorin). A scatter plot showed an inverse correlation between pI and the number of sulfates on six sugar residue structures, as shown in Figure 4*A* (one-way ANOVA, *p* = 0.0007). Moreover, a scatter plot of 8-sugar residue structures also displayed a similar inverse correlation between pI and the number of sulfates (Fig. 4*B*) (one-way ANOVA, *p* = 0.0023).

To investigate whether other features apart from the acidity of the attachment motif influence the sulfation degree, we prepared a frequency plot of the neighboring amino acids in the region from −5 to +5 of the glycosylated serine residues. The attachment sequences of different core proteins were grouped into three categories depending on the average degree of sulfation of their corresponding 6-mer structure: low sulfate (<1), intermediate sulfate (1, 2), or high sulfate (>2) (supplemental Tables S3 and S4). In line with the correlation analysis, a higher abundance of acidic residues was seen for the motif of highly sulfated 6-mer structures (supplemental Fig. S9, *A*–*C*). Notably, these acidic residues were not evenly distributed along the frequency plot but were much more prominent in the N-terminal direction, where a large portion of the sequences displayed a “D” or “E” at the −4 and −3 positions (supplemental Fig. S9*C*). Furthermore, to investigate whether additional features more distant from the glycosylation site may influence sulfation degree, we also prepared a frequency plot of the neighboring amino acids in the region from −7 to +7 of the glycosylated serine (supplemental Fig. S10, *A*–*D*). Apparently, a proline residue was relatively prominent in the N-terminal end of the CS motif of highly sulfated 6-mer structures, where 67% (6 out of 9) of the motifs displayed a proline residue in the −6 or −7 position (supplemental Fig. S10*C*). Taken together, the frequency plot analysis corroborated the statistical analysis and showed higher abundance of acidic residues for attachment motifs with highly sulfated CS structures compared to attachment motifs with intermediate or low sulfated CS structures. Moreover, certain features of the attachment sequence appear to be associated with highly sulfated CS structures, such as an acidic residue in the −4 and/or −3 position and proline in the −7 or −6 position.

### Core Protein-specific CS Structures in Human Urine

**Fig. 5:**
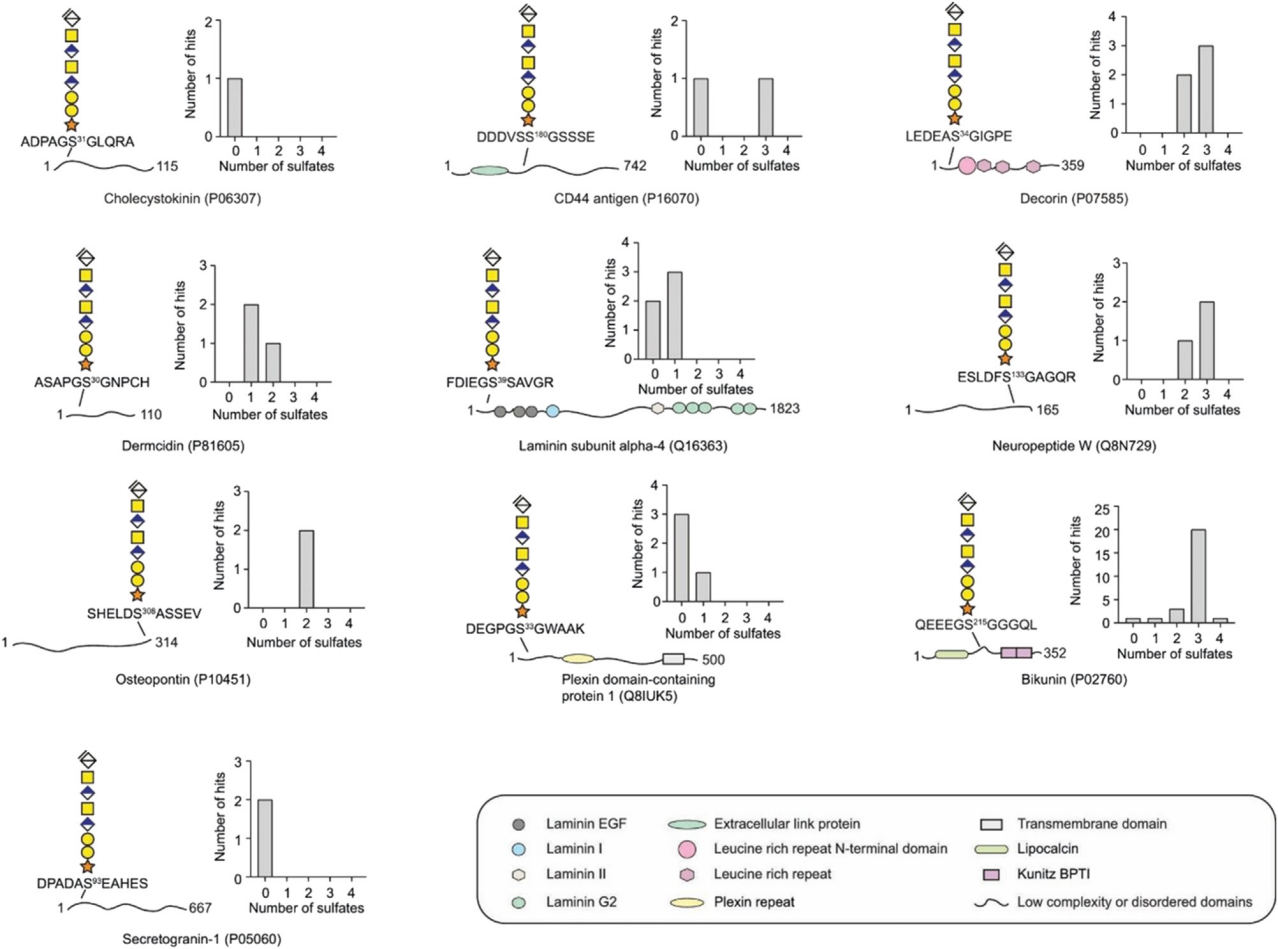
**Schematic illustration of attachment site localization and distribution of sulfate modifications of CS polysaccharides from ten different core proteins in human urine.** The scheme illustrates the core proteins identified with residual octasaccharide CS structures but without any sulfates annotated. The presence of functional domain(s) and the CS attachment motif for each core protein are shown to the *left*. Histograms based on spectral counts of annotated MS/MS spectra show the distribution of the number of sulfates of each peptide-linked CS structure. Some core proteins, such as cholecystokinin and secretogranin-1, carried residual CS structures without any sulfate modifications, while others such as bikunin, carried CS structures with mainly three sulfate modifications. The keys for various functional peptide domains are provided in the *box*.

To get a general overview of the CS glycoproteome in human urine, the identified core proteins and the level of sulfation of their peptide-associated CS structures are shown in Figure 5. For simplicity, only core protein peptides identified with residual structures of 8-mer residues (or longer) were included in the scheme. The presence of functional domain(s) and the CS-attachment motifs are schematically illustrated. Furthermore, histograms based on spectral counting of annotated MS/MS spectra (supplemental Table S1a) show the sulfate distribution (n = 0, 1, 2, 3, or 4) of the associated CS structures. Inspection of the attachment motif indicated the acidic residues are mostly found at the N-terminal side of the serine residue. Further, an examination of the domain organization and the positions of the attachment sites reveals that all CS attachment sites are located in disordered domains or domains of low complexity. However, the sulfated glycan structures had no apparent enrichment towards the N- or C-terminal domains. Furthermore, sulfated structures were found both on core proteins without any functional domains (*e.g.*, cholecystokinin and secretogranin-1) and on core proteins containing several functional domains (*e.g.*, decorin). Taken together, these results indicate that all attachment sites are found in disordered domains, but with no apparent association between the level of sulfation of the oligosaccharide and the core protein-domain organization. In conclusion, our analysis indicates that the acidity of the peptide sequences around the attachment sites is a major determinant in influencing the level of sulfation of the reducing end of CS chains in human urinary proteoglycans.

## Discussion

Structural characterization of CSPGs traditionally relies on the release of the glycan chains from the core proteins, followed by separate analysis of these two components separately. While this approach greatly facilitates the analytical procedure, it precludes site-specific information and introduces a “GAG” and a “core protein” perspective on the data interpretation. Thus, using the traditional analytical approaches, it is difficult to obtain core protein-specific information and evaluate potential structural differences between CS chains of different core proteins. Here, we performed site-specific sequencing of CS structures and evaluated potential differences in levels of sulfation of different core protein glycopeptides of human urine. The isolation and structural characterization of CSPGs from complex sample mixtures are inherently difficult due to their relatively low quantities and their heterogenic nature. The CSPGs display differences in their protein structures and/or the number of CS chains, and the heterogeneity is further compounded by the variability in CS chain lengths as well as sites and levels of sulfation. Thus, previous attempts to obtain CS structural information of specific core proteins have been performed on highly purified preparations (11, 33, 34), whereas a global approach to characterize core protein-specific structures in any given sample matrix has not been systematically attempted. To obtain sufficient quantities of CS glycopeptides for structural characterization by MS, we developed a two-step SAX procedure to enrich CS glycopeptides from human urine. The enriched CS glycopeptides were differentially incubated with only heparinase or with chondroitinase ABC (3 h or 30 s) to generate CS oligosaccharides of different chain lengths. The 3-h incubation resulted in a more or less exhaustive CS chain depolymerization, generating free disaccharides and a residual 6-mer structure still attached to the peptide (24). Proteomic database searches of the generated MS/MS data retrieved 23 previously established CSPGs and five novel CSPGs, indicating effective enrichment of CS glycopeptides with the double SAX procedure. Glycoproteomic strategies have previously identified a significant number of novel human CSPGs (24, 26), but the results of the present investigation indicate that additional CSPGs are yet to be discovered. Interestingly, two of the identified CSPGs, PGRMC1 and 2, respectively, are known as cell surface receptors, affecting steroid hormone signaling independently of the classical steroid receptors (35). We have earlier shown that several prohormones carry CS modifications and *in vitro* studies suggested that CS promotes the storage of peptide hormone in secretory granules (24, 36). Our finding that PGRMC1 and 2 are modified with CS polysaccharides suggests that CS may also have a role in steroid-hormone regulation.

A brief chondroitinase ABC incubation (30 s) resulted in partial chain depolymerization, generating glycopeptide structures that ranged from 8 to at least 18 monosaccharide residues in length. Thus, this short incubation strategy effectively reduced polysaccharide complexity while still providing structures of sufficient length to study core protein-specific CS structures. Similar to what is seen with other types of glycopeptides, the HCD-based MS/MS-fragmentation in positive ion mode generated abundant peptide and glycan fragmentations, enabling comprehensive glycopeptide characterization (37). However, as sulfate groups undergo extensive decomposition during the HCD-MS/MS-fragmentation, detailed sequencing of the sulfation pattern was not feasible (38). Nevertheless, determination of the overall level of sulfation was still possible due to the instrument’s high mass accuracy of the measured precursor ions. A relevant concern when analyzing sulfated structures by positive mode ESI-MS/MS is the potential incidence of in-source fragmentation. This phenomenon can result in sulfation losses from precursor ions, resulting in the detection of structures with apparent lower levels of sulfation compared to their native counterparts. However, our data demonstrated that bikunin and decorin displayed the same (or even higher) degree of sulfation as seen with other analytical techniques (11, 23, 39). Moreover, examination of different precursor ion intensities also demonstrated limited sulfate loss (supplemental Fig. S11, *A*–*C*), supporting the notion of limited in-source fragmentation in this analytical system. Taken together, we conclude that positive mode ESI-HCD-MS/MS analysis is an effective strategy to determine the overall level of sulfation of CS-glycopeptides.

Moreover, while some GAG–protein interactions require a strict oligosaccharide sulfation sequence, other interactions show a sliding scale of specificities and affinities that often relate to the sulfation level of the polysaccharides (40, 41, 42). Thus, determining the overall level of sulfation (as shown here) may provide sufficient information for investigating biologically relevant CS–protein interactions of lower specificities and affinities. Of note, for highly specific GAG–protein interactions, negative mode MS/MS analysis is likely required for obtaining detailed structural information of the GAG structures. However, as negative mode generally does not provide effective peptide fragmentation, the combined use of positive- and negative-mode analyses may assist in obtaining comprehensive core protein site-specific sequencing of CS chains.

Xylose phosphorylation is an important linkage region modification and is considered to function as a molecular switch to regulate GAG biosynthesis (43). For instance, xylose dephosphorylation has been regarded as a requirement for chain polymerization initiation and for efficient elongation of the disaccharide units in the CS chain (32). Surprisingly, we found phosphate modifications on xylose residues of polysaccharides of 8- and 10-monosaccharide residues in length, indicating that the removal of phosphate groups may not be strictly required for the immediate chain extension as was previously perceived. These findings warrant further functional studies of how xylose phosphate modifications regulate GAG biosynthesis.

How the chain length, sequence, and pattern of sulfation of a GAG chain is determined is regarded as an essential question in modern cell biology (44). GAG biosynthesis is considered, similar to all complex glycan biosynthesis, as a non-template-driven process, dependent on the cell- and tissue-specific expression profiles of the biosynthetic enzymes. The focus has been on GAG biosynthesis rather than on proteoglycan biosynthesis and based on such a conceptual notion, all proteoglycans would show similar (or even identical) GAG structures if they originated from the same cell type, and would display different structures only if they originate from different cell types (45). However, this concept is probably an oversimplification as reports indicate that certain core protein domains may indeed directly influence their GAG biosynthesis and structure (14, 15, 16, 17, 45). Of note, acidic residues surrounding the attachment site of thrombomodulin were shown to be indispensable for CS polymerization and its resulting fine structure (16). Our general finding that the acidity of the attachment motif correlated with the CS level of sulfation supports the notion that core protein characteristics may be of importance for CS modifications. Although the underlying mechanisms are yet unclear, it may suggest that certain CS sulfotransferases display higher affinity towards acidic peptide sequences, thereby increasing the likelihood of introducing sulfate groups on the proximal part of the CS chain. It should be noted that, although all identified 6-mer and 8-mer glycopeptides were included in the statistical analyses, not all core proteins conform to this correlation. For instance, secretogranin-1 has a relatively acidic attachment motif (DPADASEAHES; pI 3.91) but is consistently found without sulfate modifications (supplemental Table S1a). However, inspection of the attachment motif shows that secretogranin-1 lacks a “-SG-,“ or a “-SA-“ sequence, which are the preferred attachment site characteristics for the xylosyltransferases (9). Whether this “atypical” attachment site negatively affects the sulfation level remains to be determined. If so, an algorithm that combines the acidity of the attachment motif and the attachment sequence may better predict the level of sulfation of that site-specific CS chain.

Interestingly, chondroitin 6-*O*-sulfotransferase-1 (C6ST-1) transfers a sulfate group to the C-6 of the GalNAc residues, and is also involved in the transfer of a sulfate group to C-6 of the Gal residue in the linkage region; for efficient C6ST-1 sulfation of the Gal residue, the linkage region needs to be connected to a core protein, suggesting that the transferase indeed interacts with the peptide sequence (46). Whether C6ST-1, or any of the other CS sulfotransferase, shows specificity for acidic peptide sequences remains to be determined (46). If such acidic binding preference could be established, this would be similar to that of xylosyltransferase I which preferably binds peptides where serine residues are flanked by acidic amino acid residues (9).

The finding that the acidity of the attachment motif correlated with the CS level of sulfation and phosphorylation is based on our observations of residual 6-mer and 8-mer structures, respectively. Whether this correlation is also true for more distal parts of the chain remains to be determined, although it is not inconceivable that this might be the case, at least to some extent. The extended GAG biosynthesis is perceived to occur through the concerted action of multiple enzymes, generating polysaccharides with complex, yet defined, structures (47, 48). Based on such a notion, efficient recruitment of sulfotransferases at the initiation of the chain biosynthesis may promote efficient biosynthesis along the entire chain, potentially leading to higher degrees of sulfate modifications. As mentioned earlier, it is also known that the phosphate modifications of xylose, carried out by a specific kinase, do affect the initial biosynthesis of the CS chains (32, 43). However, whether these enzymes are also dependent on the acidic amino acid sequence around the glycosite or whether other interacting partners are also involved in the regulation of the overall biosynthesis of the CS chains remains to be shown.

Of importance, however, the difference in the level of sulfation of the different CSPGs, as shown here, might not only be related to the acidity of the attachment motif but may also be influenced by their cellular origin; human urine contains CSPGs derived from multiple cell types, each with different expression-profiles of their biosynthetic enzymes. For example, a cell with high expression of sulfotransferases would presumably produce CSPGs with relatively high CS sulfation levels. However, although the CSPGs in this study originate from different tissues and cell types, the differences observed in their levels of sulfation of CS chains still display a biological correlation to the acidity of the attachment sites of the core proteins irrespective of their origin. Thus, potential differences in sulfotransferase expression between different cells cannot by itself explain the observed correlation (acidic motif *versus* sulfation level) but would rather obscure this correlation. The same is probably true also for hydrolyzing enzymes, for example sulfatases or phosphatases, appearing differently in different tissues and body fluids such as urine. It is likely that both core protein-specific determinants and cellular-specific expression profiles influence the final CS chain and that the two modes are not mutually exclusive. Future studies are needed to clarify this aspect, possibly by using the method presented herein to analyze different CSPGs from different cell types and ideally also including site-specific mutagenesis of acidic amino acids surrounding the attachment sites (*e.g.*, ‘D’ to ‘A’) of various core proteins. Such a study would assist in delineating the contribution of core protein-specific determinants *versus* cellular expression profiles for the final CS structure. Moreover, while we did not identify any additional core protein features that appeared to influence the CS structure (Fig. 5), our study does not exclude the possibilities of additional, yet unexplored, core protein characteristics. Future structural and functional studies from which additional tissular, cellular, subcellular enzymatic, and core protein-specific structural information may be collected, could shed light on these questions.

In conclusion, information about how CS structures vary between different core proteins is presently relatively limited. This gap in knowledge relates in part to the vast structural heterogeneity of CSPGs and the few methods at hand that provides integrated GAG and protein structural information. To this end, the novel glycoproteomic approach described here enables site-specific sequencing of partly depolymerized CS structures with chains extending up to 18 monosaccharide residues. The results suggest that the acidity and the sequence of the amino acids neighboring the attachment motif together encode for higher levels of sulfation in the non-reducing end of the CS polysaccharide, including the linkage region. The method will likely become useful for future studies of structural-functional relationships of CSPGs in cellular physiology.

## Data Availability

The mass spectrometry proteomics data have been deposited to the ProteomeXchange Consortium *via* the PRIDE (49) partner repository with the dataset identifier PXD037990. Reviewer account details: **Username:** reviewer_pxd037990@ebi.ac.uk. **Password:** xUhgLqr2.

## Ethical Permission

The use of de-identified human samples for method development is in agreement with Swedish law and was formally permitted by the Head of the Clinical Chemistry Laboratory, Sahlgrenska University Hospital, Gothenburg, Sweden.

## Supplemental Data

This article contains supplemental data (30, 50).

## Conflict of interest

The authors declare that they have no known competing financial interests or personal relationships that could have appeared to influence the work reported in this paper.
