# Supplementary material for: Mapping the Human Chondroitin Sulfate Glycoproteome Reveals an Unexpected Correlation Between Glycan Sulfation and Attachment Site Characteristics
Source: Mol Cell Proteomics. 2023 Jul 14;22(8):100617. doi: 10.1016/j.mcpro.2023.100617 (PMC10424144; doi:10.1016/j.mcpro.2023.100617)
Supplement: Supplemental Information [file mmc1.docx]

**SUPPLEMENTARY INFORMATION**

**Mapping the human chondroitin sulfate glycoproteome reveals an unexpected correlation between glycan sulfation and attachment site characteristics**

Fredrik Noborn^1^, Jonas Nilsson^2^, Carina Sihlbom^2^, Mahnaz Nikpour^1^, Lena Kjellén^3^, Göran Larson^1,4^

^1^ Department of Laboratory Medicine, Institute of Biomedicine, Sahlgrenska Academy, University of Gothenburg, Gothenburg, Sweden.

^2^Proteomics Core Facility, Sahlgrenska Academy, University of Gothenburg, Sweden

^3^Department of Medical Biochemistry and Microbiology, Uppsala University, Uppsala, Sweden

^4^Laboratory of Clinical Chemistry, Sahlgrenska University Hospital, Gothenburg, Sweden

**Running title**: Chondroitin sulfation and attachment site characteristics

**Keywords**: glycosaminoglycans; chondroitin sulfate, proteoglycans; glycoproteomics; glycopeptides

**
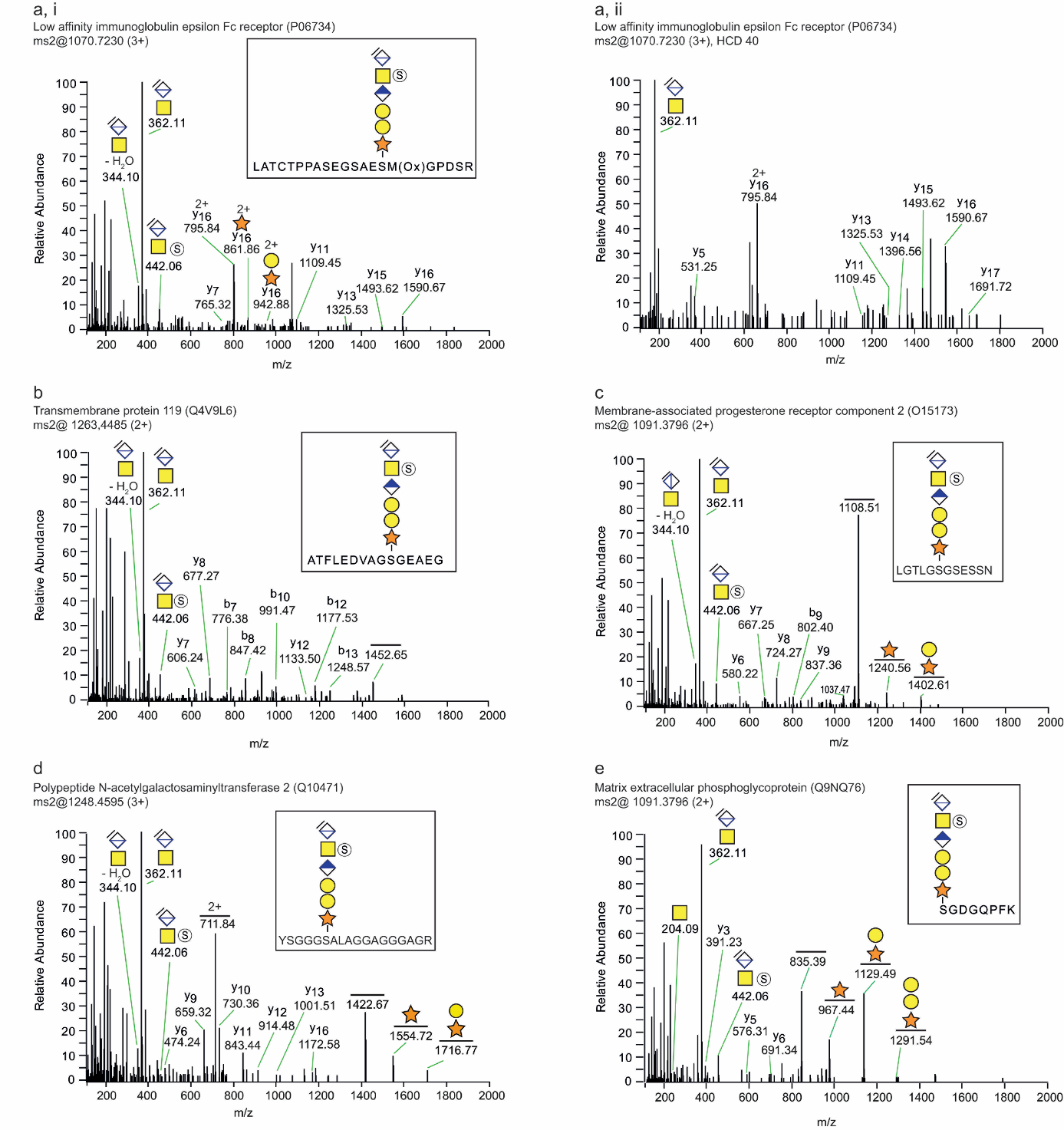
**

Supplementary Figure 1**. Fragment mass spectra of five novel CSPGs identified in this study.** Fragmentation of the precursor ions at HCD 30 (**a-e**) generated several peptide and glycosidic fragment ions, including the diagnostic GAG-fragment ion at *m/z* 362.11. This fragment corresponds to the terminal dehydrated disaccharide [HexA(-H_2_O)GalNAc+H]^+^, which is generated upon chondroitinase ABC hydrolysis of the CS chains. Fragmentation at HCD 40 of the precursor ion for low affinity immunoglobulin epsilon Fc receptor (**a, ii**) provided additional peptide fragment ions, thus further strengthen the identity of the peptide sequence.

**
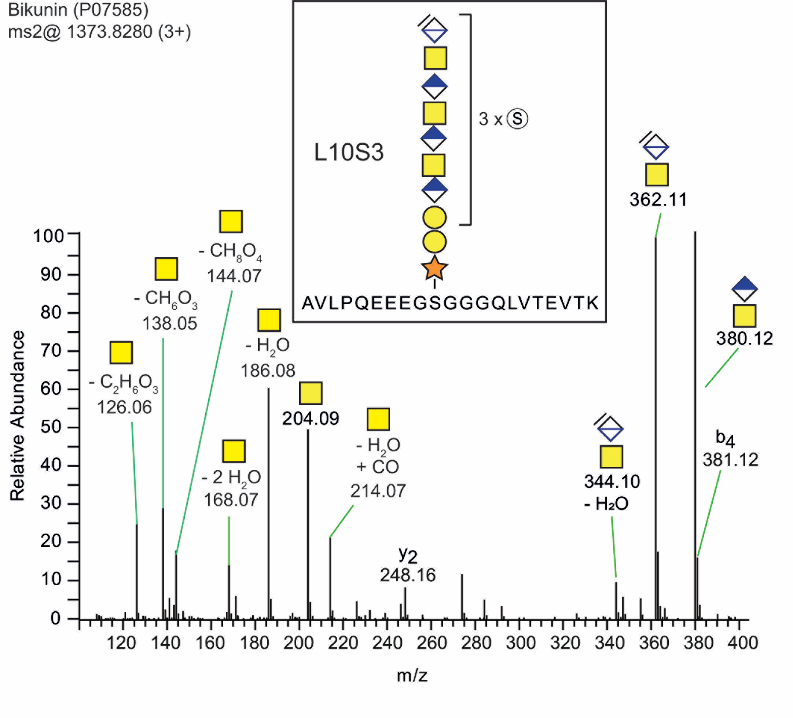
**

Supplementary Figure 2**. Identification of GalNAc-derived oxonium ions.** Detailed examination of the low mass range (*m/z* 100-400) enabled the identification of several GalNAc-derived oxonium ions in all CS glycopeptide mass spectra. Such ions were the result of H_2_O losses (*m*/*z* 168.1 and *m*/*z* 186.1) and saccharide decompositions (*m*/*z* 126.1, *m*/*z* 138.1, *m*/*z* 144.1, and *m*/*z* 214.1). As an example, an enlarged view of mass spectrum of the bikunin glycopeptide (*m/z* 1373.8280; 3+) in Figure 2**a** is shown.

**
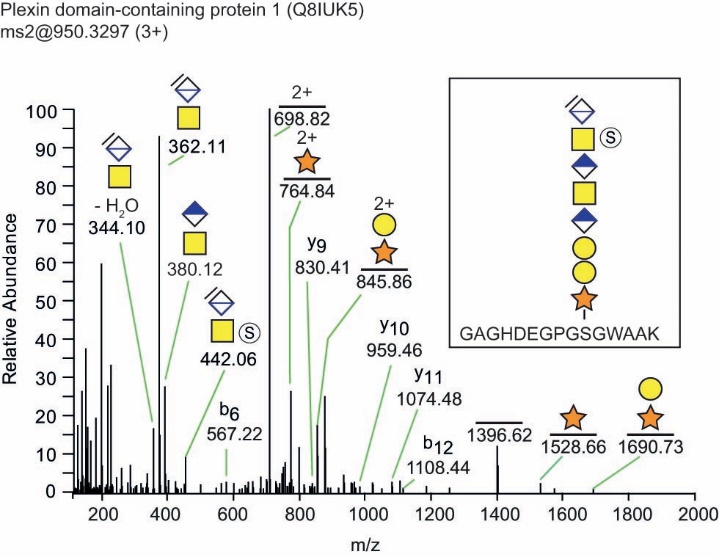
**

Supplementary Figure 3**. Plexin domain-containing protein 1 carried a residual 8-sugar structure with one sulfate modification.** MS/MS fragment spectrum of plexin domain-containing protein 1 (Q8IUK5) (*m/z* 950.3297; 3+). The glycopeptide was composed of the peptide backbone (GAGHDEGPGSGWAAK) associated with an 8-sugar modification and 1 sulfate modification (SO_3_^-^). The sulfate modification was positioned at the subterminal GalNAc residue, as shown by the presence of the fragment ion at *m/z* 442.06-ion, which corresponds to the terminal dehydrated disaccharide with one sulfate group [HexA(-H_2_O)GalNAc+ SO_3_+H]^+^.

**
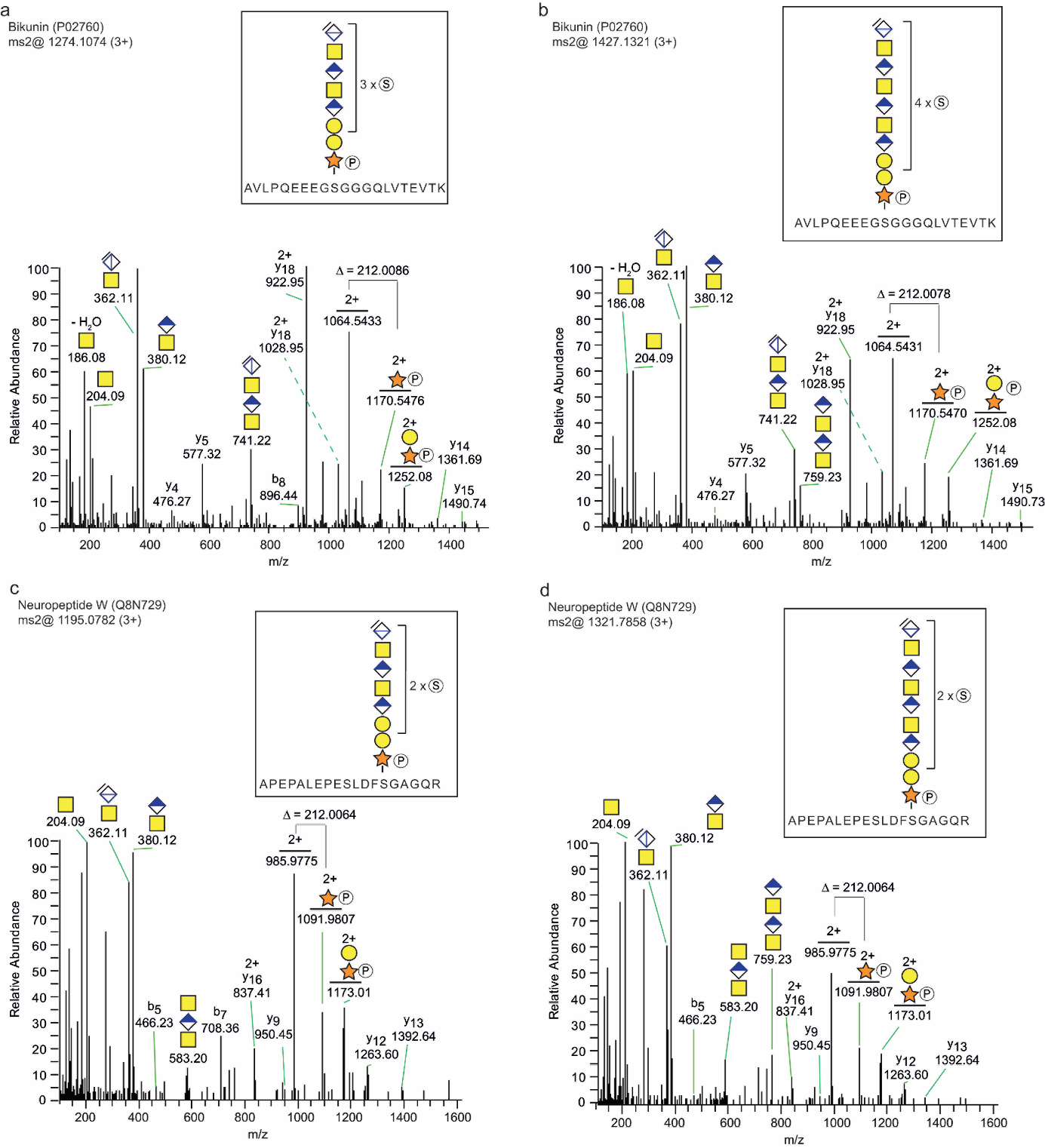
**

Supplementary Figure 4**. Bikunin- and neuropeptide W-associated CS polysaccharides with phosphate modifications.** Fragment mass spectra of bikunin (**a-b**) and neuropeptide W (**c-d**) carrying 8- and 10 sugar residual structures with sulfate- and phosphate modifications. (**a-b**) Detailed examination of the bikunin spectra revealed mass shifts of 212.0086 Da (**a**) and 212.0076 Da (**b**), respectively, corresponding to the mass of a xylose and a phosphate modification [xylose (132.0423 Da) + phosphate group (79.9663 Da) = 212.0086 Da). (**c-d**) The neuropeptide W spectra displayed similar mass shifts of 212.0064 Da in (**c**) and (**d**), also demonstrating the presence of a xylose and a phosphate modification.

**
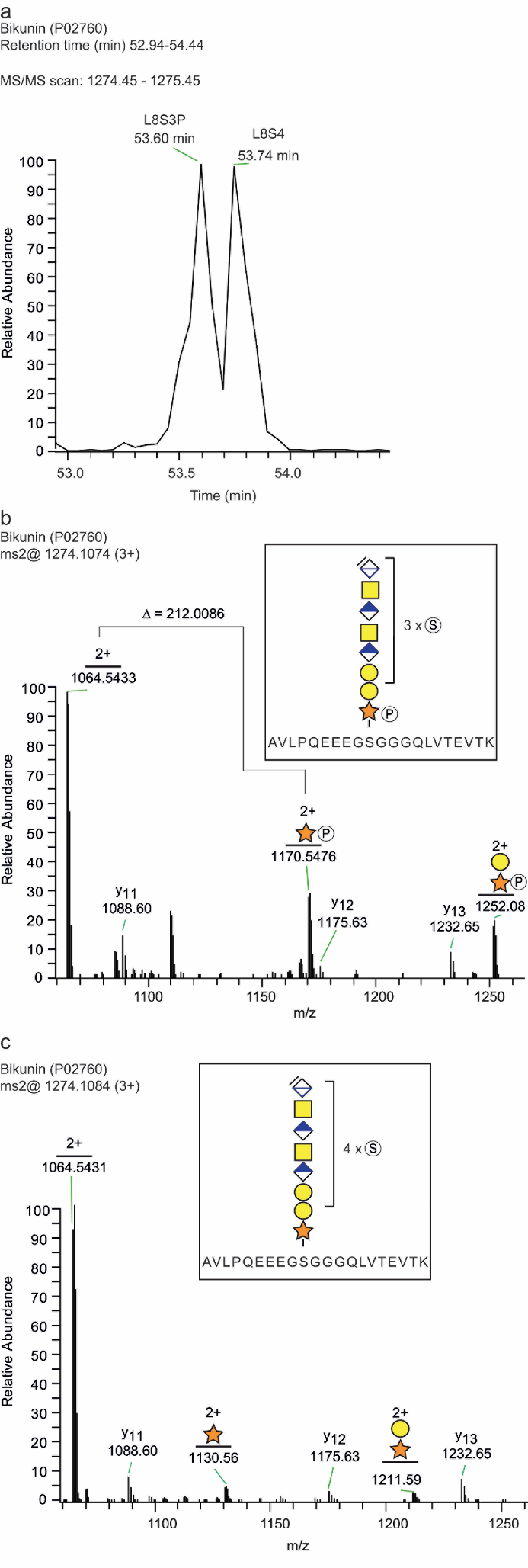
**

Supplementary Figure 5**. Bikunin associated structures with and without phosphate modification.** (**a**) An extracted ion chromatogram of the bikunin precursor ions (*m/z* 1274.45 – 1275.45) shows chromatographic separation between the two structural variants. The phosphate-modified 8-sugar structure (L8S3P) elutes earlier with a maximum at 53.60 min, whereas the non-phosphorylated structure (L8S4) elutes at 53.74 min. The extracted ion current represents the second isotopic peak, as this constitutes the largest peak of the isotopic distribution. **(b-c)** Detailed examination of the fragment mass spectrum between *m/z* 1000-1300 for bikunin 8-sugar structures with (**b**) and without (**c**) phosphate modifications. **(b)** Phosphate modification on the xylose residue resulted in a prominent fragment ion (peptide+Xyl+PO_3_, *m* /*z* 1170.5476; 2+). A mass shift of 212.0086 Da was observed between *m/z* 1170.5476 and *m / z* 1064.5433, corresponding to the mass of a xylose and a phosphate modification [xylose (132.0423 Da) + phosphate group (79.9663 Da) = 212.0086 Da). (**c**) Sulfate modifications on the galactose residues does not result in any prominent fragment ions in positive mode analysis.

**
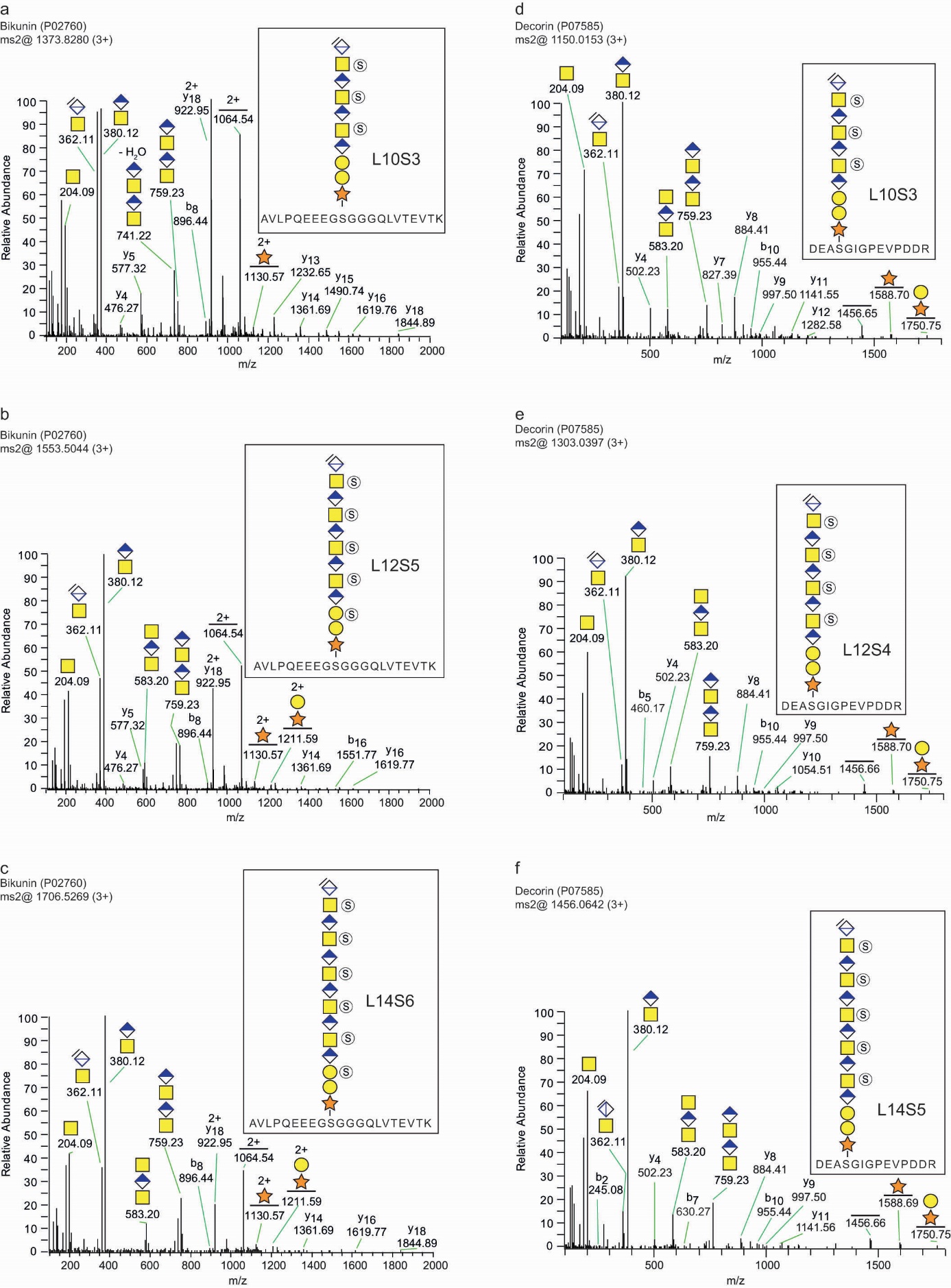
**

Supplementary Figure 6**. Site-specific CS analyses of decorin and bikunin suggest a uniform sulfate distribution along the chains.** Fragment mass spectra of bikunin (**a-c**) and decorin (**c-f**) carrying sulfated CS structures, ranging from 10-sugar to 14-sugar residues in length. The spectra show that the stepwise increase of GalNAc-residues is accompanied with one additional sulfate modification (SO_3_^-^).


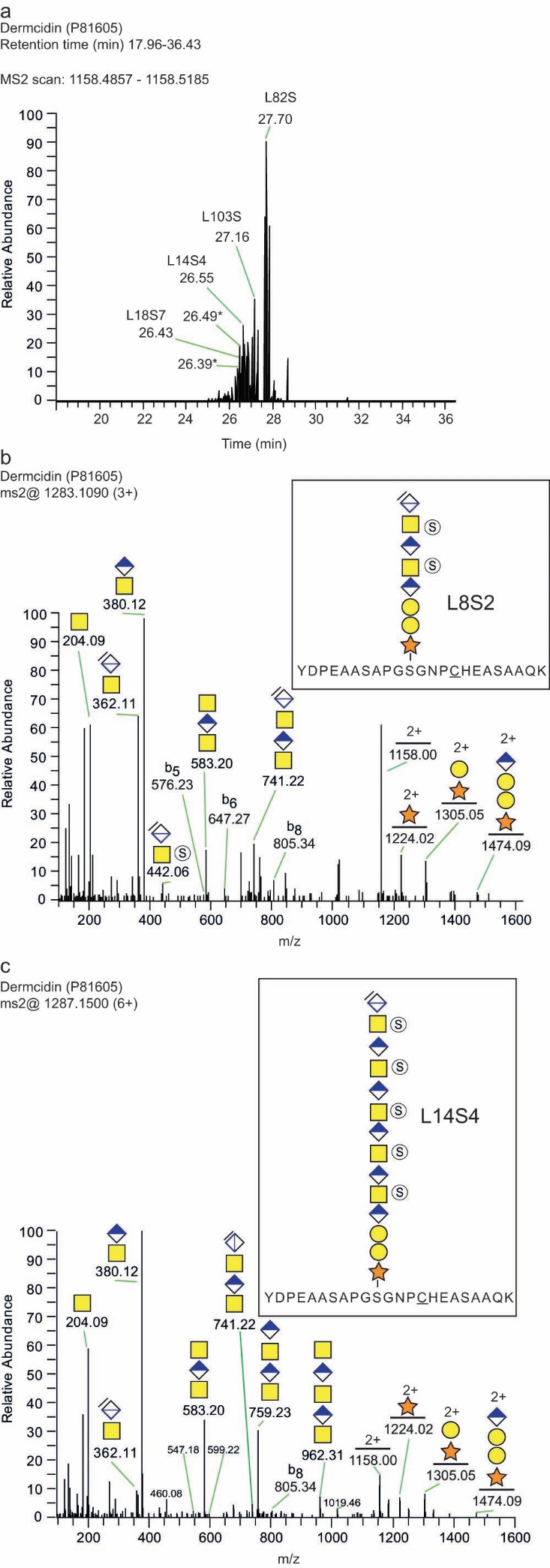


Supplementary Figure 7**. Site-specific CS analysis of dermcidin suggests a uniform sulfate distribution. (a)** An extracted-ion current chromatogram of the dermcidin peptide ion (*m/z* 1158.4857-1158.5185) show multiple forms of dermicin-associated CS polysaccharides, ranging from 8 to 18 sugar residues in length. Additional glycoforms were observed at 26.49 min and 26.39 min (denoted with asterisks), but their exact identity could not be determined due to abundant adduct formation. The extracted ion current represents the second isotopic peak, as this constitutes the largest peak of the isotopic distribution. (**b**) MS/MS fragment spectrum of dermcidin (P81605) (*m/z* 1306.1312; 3+). The CS-glycopeptide was composed of the peptide backbone (YDPEAASAPGSGNPCHEASAAQK) with one carbamidomethyl (CAM) modification associated with an 8-sugar modification and 2 sulfate modification (SO_3_^-^). (**c**) MS/MS spectrum showing dermcidin with 14-sugar residues and 4 sulfate modifications.


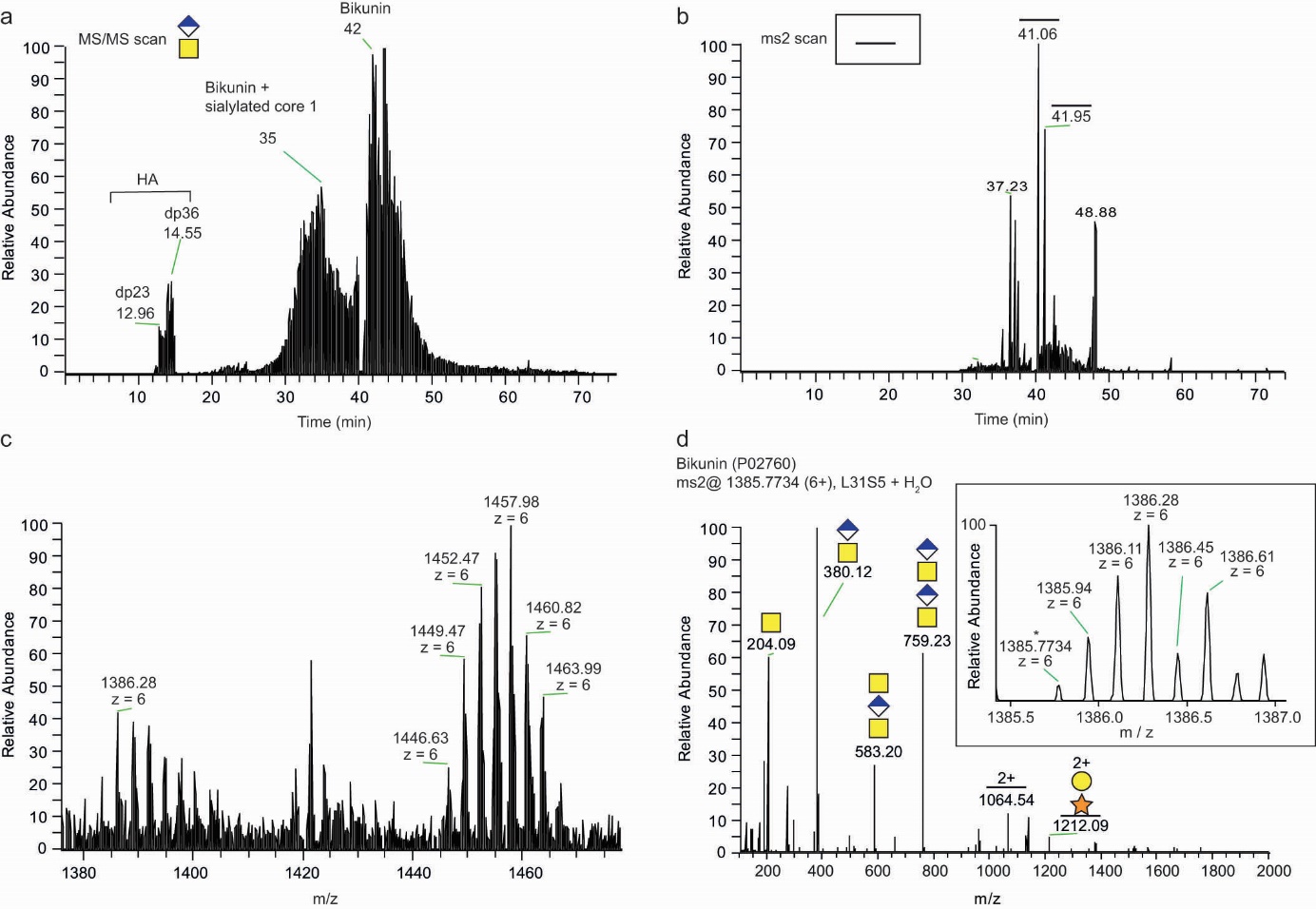


Supplementary Figure 8**. Analysis of full-length bikunin structures. (a)** An extracted-ion current chromatogram of MS/MS spectra which have been filtered for the presence of the m/z 380.12 diagnostic ion, representing the disaccharide ion [HexAGlcNAc]^+^, revealed several 380-related peaks eluting at various positions. Hyaluronic acid (HA) was identified at ~ 12-16 min and was found of different lengths, including dp23 and dp36 (dp; degree of polymerization, i.e. the number of monosaccharide residues). Full-length bikunin eluted as two major chromatographic peaks at ~35 and ~42 min, representing glycopeptides composed of variants of the trypsin digested bikunin peptide sequence associated with full-length CS structures. Notably, the CS-glycopeptide chromatographic peak eluting at ~35 min also contained a sialylated core 1 O-glycan on a separate Ser/Thr residue (1). (**b**) An extracted-ion current chromatogram of MS/MS spectra which have been filtered for the bikunin peptide (*m/z* 1065.01-1065.07; AVLPQEEEGSGGGQLVTEVTK), revealed a similar elution pattern as the chromatogram of the 380-related peaks. The filtered peak corresponds to the second isotopic peak, which is the largest peak in the isotopic distribution for the doubly charged peptide ion (**c**) A MS1 spectrum at *m/z* 1350-1480 (collected between 44.00 min to 45.02 min) showed several large precursor ions, representing variants of the full-lengths bikunin. However, determining their exact identity was difficult in many cases due to high structural heterogeneity. (**d**) MS/MS spectra of bikunin (*m/z* 1385.7734; 6+) representing 31-sugars with five sulfate modification and one water adduct (L31S5 + H_2_O). (Insert) MS1 of the molecular ion showing the monoisotopic peak (*) used for calculation of the mass accuracy.

**
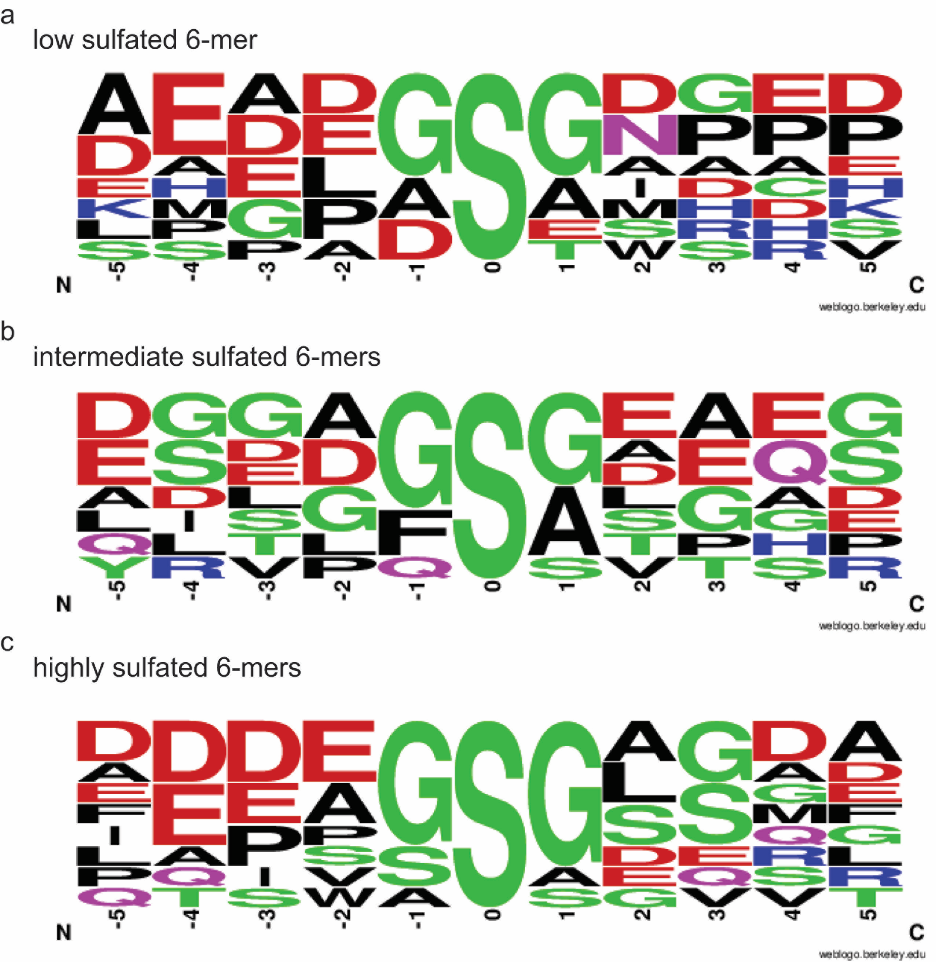
**

Supplementary Figure 9**. Sequence motif analysis of CS attachment sites of different degree of sulfation.** A statistical analysis of aligned sequences of low sulfated (**a**), intermediate sulfated (**b**) and highly sulfated (**c**) attachment sites. Sequence logos show the frequency of each amino acid in the region from -5 to +5 of the glycosylated serine residues. The figures were prepared using WebLogo (2).

**
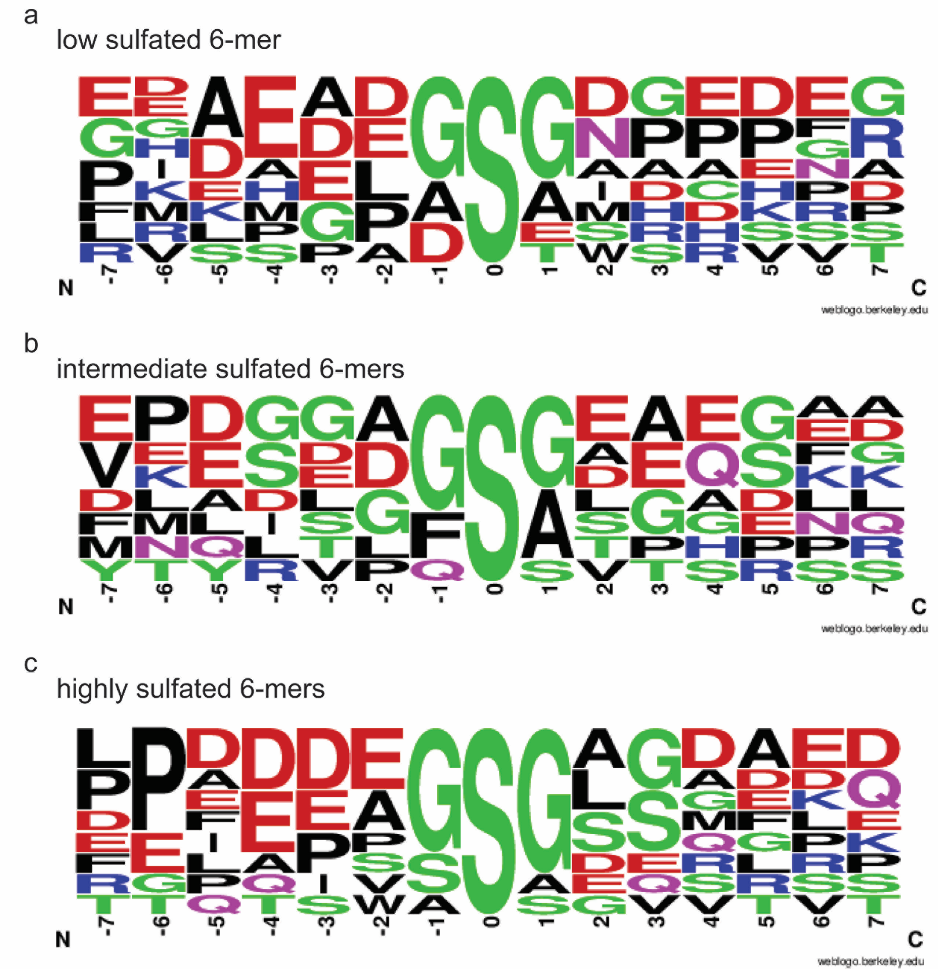
**

Supplementary Figure 10**. Definition of an attachment motif which encodes for highly sulfated 6-mer CS structures.** A,statistical analysis of aligned sequences of low sulfated (**a**), intermediate sulfated (**b**) and highly sulfated (**c**) attachment sites. Sequence logos show the frequency of each amino acid in the region from -7 to +7 of the glycosylated serine residues. The figures were prepared using WebLogo (2).


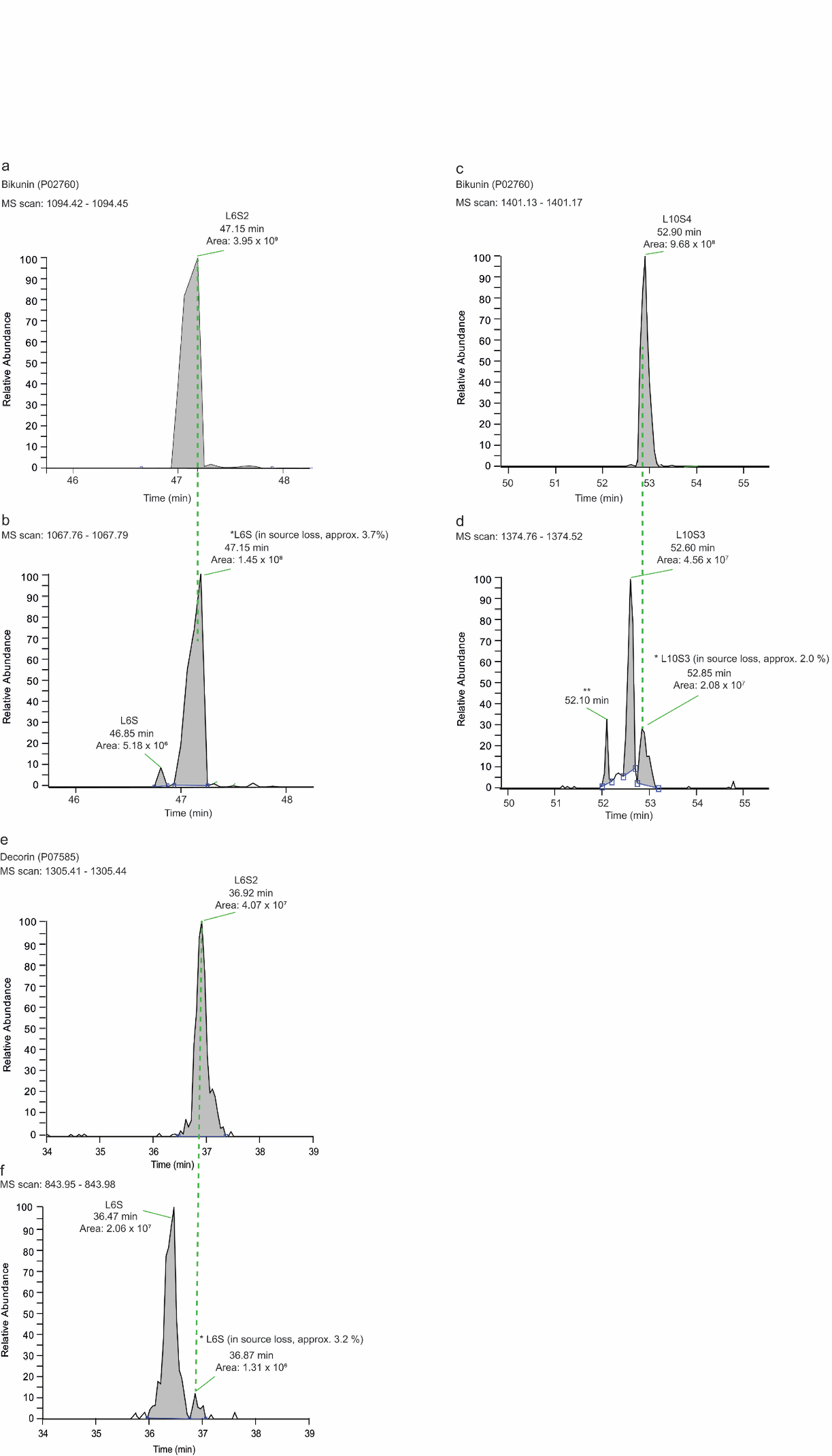


Supplementary Figure 11**. Extracted ion chromatograms of glycopeptide precursor ion intensities indicate limited in-source sulfate losses.** (**a**) Extracted ion chromatogram of the precursor ion L6S2 (*m/z* 1094.42-1094.45) of bikunin (P02760) shows a distinct chromatographic peak at 47.15 min (area intensity 3.95 x10^9^). (**b**) Extracted ion chromatogram of the precursor ion L6S (*m/z* 1067.76-1067.79) of bikunin shows two peaks with different intensities. An ion (*L6S), with a peak area intensity of 1.45 x 10^8^, is observed at 47.15 min which co-elutes with the L6S2 ion in **a** (the peaks are aligned with a dashed line). Given that the ions co-elute, the *LS6 ion is deduced to be the result of in-source sulfate loss from L6S2, i.e., L6S2-S. The peak area intensity of the L6S2-S ion is approximately 3.7 % of the L6S2 peak in **a**, i.e. (1.45 x 10^8^/3.95 x10^9^)*100, indicating limited in-source sulfate loss. The less intense peak in **b** occurring at 46.85 min (peak area 5.18 x 10^6^ ) corresponds to the native L6S ion of bikunin. (**c**) Extracted ion chromatogram of the precursor ion L10S4 (*m/z* 1401.13-1401.17) of bikunin shows a distinct chromatographic peak at 52.90 min (area intensity 9.68 x10^8^). (**d**) Extracted ion chromatogram of the precursor ion of bikunin L10S3 (*m/z* 1374.76-1374.52) shows several peaks with varying intensities. One ion (*L10S3) observed at 52.85 min (area intensity 2.08 x 10^7^) co-elutes with the L10S4 ion in **c**, and is deduced to be the result of in-source loss from L10S4, i.e. L10S4-S. The intensity of the L10S4-S ion is approximately 2.0 % of L10S4. The major peak in **d** occurring at 52.60 min (peak area 4.56 x 10^7^) corresponds to the native L10S3 of bikunin. The identity of the ion eluting at 52.10 min (**) could not be settled but may be a structural isomer to the dominating L10S3 structure. (**e**) Extracted ion chromatogram of the precursor ion L6S2 (*m/z* 1305.41-1305.44) of decorin (P07585) shows a distinct chromatographic peak at 36.92 min (area intensity 4.07 x10^7^). (**f**) Extracted ion chromatogram of the precursor ion L6S (*m/z* 843.95-843.98) of decorin shows two peaks with varying intensities. One ion (*L6S) observed at 36.87 min (area intensity 1.31 x 10^6^) co-elutes with the L6S2 ion in **e**, and is deduced to be the result of in-source sulfate loss, i.e. L62S-S. The intensity of L62S-S is approximately 3.2 % of native L6S2. The dominating peak in **e,** occurring at 36.47 min (peak area 2.06 x 10^7^), corresponds to the native L6S ion of decorin.

**References**

1. Gomez Toledo, A., Nilsson, J., Noborn, F., Sihlbom, C., and Larson, G. (2015) Positive Mode LC-MS/MS Analysis of Chondroitin Sulfate Modified Glycopeptides Derived from Light and Heavy Chains of The Human Inter-alpha-Trypsin Inhibitor Complex. *Mol Cell Proteomics* **14**, 3118-3131

2. Crooks, G. E., Hon, G., Chandonia, J. M., and Brenner, S. E. (2004) WebLogo: a sequence logo generator. *Genome Res* **14**, 1188-1190
